# Common conditions associated with hereditary haemochromatosis genetic variants: cohort study in UK Biobank

**DOI:** 10.1136/bmj.k5222

**Published:** 2019-01-16

**Authors:** Luke C Pilling, Jone Tamosauskaite, Garan Jones, Andrew R Wood, Lindsay Jones, Chai-Ling Kuo, George A Kuchel, Luigi Ferrucci, David Melzer

**Affiliations:** 1 Epidemiology and Public Health Group, University of Exeter Medical School, RD&E Wonford, Exeter EX2 5DW, UK; 2 Genetics of Complex Traits Group, University of Exeter Medical School, Exeter, UK; 3 Biostatistics Center, CT Institute for Clinical & Translational Science, University of Connecticut Health Center, Farmington, CT, USA; 4 Center on Aging, University of Connecticut Health Center, Farmington, CT, USA; 5 National Institute on Aging, Baltimore, MD, USA

## Abstract

**Objective:**

To compare prevalent and incident morbidity and mortality between those with the *HFE* p.C282Y genetic variant (responsible for most hereditary haemochromatosis type 1) and those with no p.C282Y mutations, in a large UK community sample of European descent.

**Design:**

Cohort study.

**Setting:**

22 centres across England, Scotland, and Wales in UK Biobank (2006-10).

**Participants:**

451 243 volunteers of European descent aged 40 to 70 years, with a mean follow-up of seven years (maximum 9.4 years) through hospital inpatient diagnoses and death certification.

**Main outcome measure:**

Odds ratios and Cox hazard ratios of disease rates between participants with and without the haemochromatosis mutations, adjusted for age, genotyping array type, and genetic principal components. The sexes were analysed separately as morbidity due to iron excess occurs later in women.

**Results:**

Of 2890 participants homozygous for p.C282Y (0.6%, or 1 in 156), haemochromatosis was diagnosed in 21.7% (95% confidence interval 19.5% to 24.1%, 281/1294) of men and 9.8% (8.4% to 11.2%, 156/1596) of women by end of follow-up. p.C282Y homozygous men aged 40 to 70 had a higher prevalence of diagnosed haemochromatosis (odds ratio 411.1, 95% confidence interval 299.0 to 565.3, P<0.001), liver disease (4.30, 2.97 to 6.18, P<0.001), rheumatoid arthritis (2.23, 1.51 to 3.31, P<0.001), osteoarthritis (2.01, 1.71 to 2.36, P<0.001), and diabetes mellitus (1.53, 1.16 to 1.98, P=0.002), versus no p.C282Y mutations (n=175 539). During the seven year follow-up, 15.7% of homozygous men developed at least one incident associated condition versus 5.0% (P<0.001) with no p.C282Y mutations (women 10.1% *v* 3.4%, P<0.001). Haemochromatosis diagnoses were more common in p.C282Y/p.H63D heterozygotes, but excess morbidity was modest.

**Conclusions:**

In a large community sample, *HFE* p.C282Y homozygosity was associated with substantial prevalent and incident clinically diagnosed morbidity in both men and women. As p.C282Y associated iron overload is preventable and treatable if intervention starts early, these findings justify re-examination of options for expanded early case ascertainment and screening.

## Introduction

Hereditary haemochromatosis is an iron overload disease and is the most common genetic condition in people of European descent. Hereditary haemochromatosis type 1 is predominantly attributable to two *HFE* gene mutations, with 95% of affected people having the p.C282Y (p.Cyst282Tyr) mutation and 4% having the p.C282Y/p.Hist63Asp compound heterozygote genotype.^1^ The p.C282Y mutation alters a key amino acid, which in adults impairs HFE protein signalling and leads to reduced expression of hepcidin mRNA, decreased plasma hepcidin levels, and excessive systemic iron accumulation.^2^

The p.C282Y variant is present in 10-15% of populations of northern European descent, with approximately 1/150 people being homozygotes^3^ and the highest genotype prevalence reported in Ireland and Britain. In the USA, the prevalence of heterozygosity (C282Y/no p.C282Y mutations) in the third National Health and Nutrition Examination Survey (NHANES III)^4^ (n=5171) was 9.5% (95% confidence interval 8.1% to 11.3%) in Non-Hispanic white people, with a lower prevalence in Non-Hispanic black people (2.3%, 1.5% to 3.4%) and Mexican-Americans (2.8%, 1.9% to 4.0%): 0.3% (0.1% to 0.8%) of studied Non-Hispanic white people were p.C282Y homozygote, with only one case each of p.C282Y homozygotes in the other two groups. Other *HFE* mutations linked to iron overload are present in other populations.

Hereditary haemochromatosis is characterised by accumulation of iron in parenchymal organs, and clinical presentation classically includes liver cirrhosis, diabetes, and changes to skin colour, but diagnosis is now commonly triggered by chronic fatigue or arthropathy.^5^ Patients with haemochromatosis are susceptible to some infections,^6^ but the p.C282Y genotype is also associated with lower low density lipoprotein cholesterol levels.^7^

Studies of close relatives of patients with p.C282Y associated haemochromatosis suggested high penetrance to clinical disease: for example, Jacobs et al^8^ studied 735 first degree relatives of 224 Dutch p.C282Y homozygotes with clinically overt haemochromatosis and found 45.7% of these family members had a haemochromatosis related diagnosis compared with 19.4% in general population controls. However, several studies in general populations have suggested much lower penetrance to clinical disease. The largest previous non-family based genetic study of haemochromatosis (HEIRS^9^) examined 62 749 women and 36 962 men (aged 25-100 years, median age 50 years) from five North American clinical centres and included 299 *HFE* p.C282Y homozygotes. Genotype associations were tested with arthritis, diabetes, liver disease, heart disease, and impotence or infertility. The only association found was between p.C282Y homozygosity and “any liver disease” in men (odds ratio 3.28, 95% confidence interval 1.49 to 7.22 compared with neither *HFE* variant), with liver disease present in 3.3% of the p.C282Y homozygote men studied. Similarly, Beutler et al^10^ studied 41 038 people (n=152 p.C282Y homozygotes) attending a health appraisal clinic in the USA and concluded that less than 1% of p.C282Y homozygotes develop “frank clinical hemochromatosis.” However, clinical penetrance is best assessed on a lifetime, or at least later life, basis, and a recent review of four cohort studies of untreated p.C282Y homozygotes (total 107 men and 129 women) suggested that 9% of male p.C282Y homozygotes eventually develop what the authors considered to be serious liver disease.^11^ Iron accumulates with advancing age, but data from large samples of older people who are homozygous for p.C282Y have been limited. Phlebotomy is effective at preventing iron overload and reducing or stabilising several but not all clinical outcomes,^5^ especially when started early. Case finding for haemochromatosis and *HFE* variants in close relatives is indicated, but general population screening is not recommended.^12^ ^13^ This is partly because of the reported low clinical penetrance to liver disease and the absence of excess mortality in major studies.^14^

We estimated associations between *HFE* p.C282Y status and common conditions potentially linked to haemochromatosis in the large United Kingdom Biobank genotyped sample. We replicated the approaches previously used in studies of *HFE* mutations in larger general samples (mainly in HEIRS^9^ and Beutler et al^10^), with some extensions. We tested genotype associations with conditions previously linked to *HFE* p.C282Y status or seen in people with haemochromatosis^15^—namely, haemochromatosis, liver disease (and liver cancer), diabetes mellitus, coronary artery disease and atrial fibrillation, arthrosis,^16^ arthritis (and hip replacements^17^), osteoporosis,^18^ tiredness,^5^ and susceptibility to infection^6^ assessed through occurrence of pneumonia. We used data on 451 243 UK Biobank community volunteers of European descent aged 40 to 70 years at baseline. We also examined associations with outcomes for the p.H63D *HFE* variant, plus the combined effect of three other small effect genetic variants influencing iron levels: rs855791 (nearest gene: *TMPRSS6*), rs7385804 (*TFR2*), and rs8177240 (*TF*).^19^

## Methods

The UK Biobank study datasets include 502 634 volunteers aged 40 to 70 years old (with small numbers to age 73), recruited through postal invitation to those who are registered with the UK National Health Service, living within 25 miles of 22 assessment centres in England, Scotland, and Wales. Baseline assessments (2006-10) included demographics, lifestyle, disease history, and physiological measurements.^20^ Participants gave informed consent for genotyping and data linkage to medical records. The volunteers tended to be healthier at baseline than the general UK population.^21^

### Genotyping and sample selection

Data were on 451 243 UK biobank participants of European descent with *HFE* p.C282Y (rs1800562) genotype information. We also investigated *HFE* p.H63D (rs1799945), as well as three iron related variants in other genes^19^ (rs855791, rs7385804, and rs8177240: see supplementary information).

### Disease ascertainment

Ascertainment of prevalent disease was by participant responses to questionnaire items on doctor diagnosed diseases at baseline, and inpatient hospital records from 1996 to baseline interview according to ICD-10 (international classification of diseases, 10th revision) codes (see supplementary table 1 and supplementary information). Diagnoses ascertained were haemochromatosis, liver conditions (any, based on ICD-10 codes), liver cancer (incident cases only), pneumonia, coronary artery disease (myocardial infarction or angina), atrial fibrillation, diabetes mellitus (predominantly type 2 but including type 1, as typing of diabetes in haemochromatosis might be unclear), osteoporosis, osteoarthritis, and rheumatoid arthritis. Numbers of p.C282Y homozygous participants with liver cancer (at baseline), diagnosed cardiomyopathy or heart failure, or reporting impotence or infertility, were too low to analyse. Participants self reported whether they were currently taking cholesterol lowering drugs and whether they had had a hip replacement; these were used in sensitivity analyses.

Self reported tiredness or lethargy in the previous two weeks at baseline in the UK Biobank study was reclassified from “not at all,” “several days,” “more than half the days,” and “nearly every day,” combining the last two categories, compared with the rest. Participants responding “do not know” or “prefer not to answer” were excluded from analyses (<3% of responders).

Follow-up was in hospital inpatient data to March 2016, national cancer registries (to September 2015), or death registration (February 2016): maximum follow-up was 9.4 years (mean 7 years). Analyses of each incident condition excluded those with each diagnosis at baseline.

### Missing data

We excluded participants without imputed genotype data (n=15 233/502 642, 3.0%) and those with imprecise imputation for p.C282Y (n=183/487 409, 0.04%). Less than 0.5% of participants had no recorded answers to questions on self reported disease diagnoses, cholesterol lowering drug use, hip replacement, or frequency of tiredness. Given the low level of missing data, we excluded participants with specific missing data from each analysis, as needed.

### Statistical analysis

We sought to replicate the approaches previously used in studies of *HFE* mutations in larger general samples (mainly in HEIRS^9^ and Beutler et al^10^), with some extensions. Logistic regression tested baseline (cross sectional) genotype condition associations, with Cox proportional hazards regression models for all cause mortality and incident conditions. Models were adjusted for age, assessment centre, population substructure using the first five principal components of genetic variation generated in participants of European descent, and genotyping microarray (Affymetrix Axiom array 90% participants, Affymetrix BiLEVE array, sharing >95% content). In sensitivity analyses for models of incident diagnoses we used Fine and Gray competing risk models, with all cause mortality as the competing risk.^22^ All analyses were performed in Stata v14.1 with functions stcox and stcrreg for Cox proportional hazards regression models and competing risk models, respectively. Function prtest was used to test for significant differences in proportions. Figures were generated in R v3.4.1 using packages metafor (v2.0) and ggplot2 (v2.2.1). An assumption of Cox’s proportional hazards (and competing risks) regression models is that the hazard ratio remains constant over time. The STATA function stphtest was used to test whether the proportional hazards assumption was violated in our models. We have followed the American Medical Association’s recommendation that P values <0.001 do not need to be specified (see the supplementary material for actual P values, often much lower).

We aimed to replicate the approaches previously used, but we did not register a specific analysis protocol before undertaking these analyses. Similarly the specific contents of measures of overall excess morbidity were not prespecified.

### Multiple testing

Our study is essentially a replication of previous studies (especially HEIRS^9^ and Beutler et al^10^), examining associations between the two main *HFE* mutations and haemochromatosis associated morbidity. Our main analyses tested 11 key outcomes, all previously implicated in the single shared underlying process of iron overload—namely, haemochromatosis, cardiovascular disease, diabetes mellitus, any liver disease (with subanalyses of liver cancer), osteoarthritis, osteoporosis, rheumatoid arthritis, pneumonia, atrial fibrillation, tiredness, and death. We performed separate analyses for men and women, based on a priori evidence of later disease onsets in women. After the main analyses including the whole group aged 40 to 70, we provided age subgroup estimates, as iron accumulation progresses with advancing age. Extensive evidence exists for associations of the studied comparisons in people with haemochromatosis or with the p.C282Y genotype, so prior probabilities were not small, although unquantified in a comparable sample. We have reported and highlighted a main summary measure (ie, one or more of the core haemochromatosis associated diseases in all the studied men and women separately) as our overall main result. Given the context of this analysis, we report simple P values without correction for multiple testing, as we tested associations with raised prior probabilities. However, we also performed Benjamini-Hochberg multiple testing correction and report the effect on the main results.

### Mendelian randomisation

To test the hypothesis that the *HFE* genotypes affect risk of incident disease by affecting iron levels, we utilised mendelian randomisation analysis methods. The mean transferrin saturation (%) (the most specific clinical measure of iron load) was used for each of the five *HFE* genotype groups (p.C282Y homozygotes, p.C282Y heterozygotes, p.C282Y/p.H63D compound heterozygotes, p.H63D alone, and those without either mutations), as reported by Allen et al.^23^ We used R (v3.5.1) package BSDA (v1.2.0) and function tsum.test to determine the difference in transferrin saturation between each genotype and the group with no p.C282Y mutations (see supplementary table 10). For each of the six main incident outcomes (haemochromatosis, liver disease, liver cancer, osteoarthritis, osteoporosis, and diabetes) we performed Cox’s proportional hazards regression models, using the same *HFE* genotype combinations as in Allen 2008 (see supplementary table 10). R package MendelianRandomisation (v0.3.0) tested whether the association between *HFE* genotype and incident outcomes could be caused by differences in transferrin saturation owing to genotype. The inverse variance weighted result was the primary method, and the weighted median and MR-Egger methods were used as sensitivity analysis to check for consistency and bias.

### Patient and public involvement

Patients and participants were and are extensively involved in the UK Biobank study itself. We used anonymised data that were already collected and therefore no patients were involved in setting the research question or the outcome measures. No patients were asked to advise on interpretation or writing up of results. UK Biobank notified participants of relevant health related findings in the baseline assessment, but there is no individual notification of subsequent findings, including in the genotyping. We do plan to disseminate the general results of the research to study participants and to the relevant patient community.

## Results

Analyses included the 451 243 UK Biobank participants of European ancestry aged 40 to 70 years at baseline, of whom 2890 were homozygous for p.C282Y (0.6%, or 1 in 156) and 64 444 (14.3%) heterozygous for p.C282Y: the remaining group with no p.C282Y mutations present, irrespective of p.H63D status, formed the key comparison group for the main analyses (n=383 909, 85.1%).

Haemochromatosis (hereditary not specified in the diagnostic data available) was diagnosed (table 1) at baseline in 7.3% (210/2890) of the p.C282Y homozygotes overall (versus 0.02% of those with no p.C282Y mutations), increasing to 15.1% (437/2890) by end of follow-up (versus 0.04% of those with no p.C282Y mutations). Rates of haemochromatosis diagnosis were higher in men: 156 of 1294 p.C282Y male homozygotes (12.1%, 95% confidence interval 10.3% to 14.0%; table 1) at baseline, increasing to 281 (21.7%, 19.5% to 24.1%) by end of follow-up. In 1596 women, haemochromatosis diagnoses were present at baseline in 54 (3.4%, 2.6% to 4.4%), increasing to 156 (9.8%, 8.4% to 11.2%) during follow-up.

**Table 1:** Characteristics of sample, stratified by p.C282Y genotype (homozygotes, heterozygotes, and no p.C282Y mutations, sex and age

Characteristics	Men				Women		
	Homozygotes	Heterozygotes	No p.C282Y mutations*		Homozygotes	Heterozygotes	No p.C282Y mutations*
All ages (40-70 years):							
No (%)	1294 (0.6)	29 536 (14.3)	175 539 (85.1)		1596 (0.65)	34 908 (14.3)	208 370 (85.1)
Mean (SD) age (years)	56.85 (8.18)	57.01 (8.12)	57.00 (8.11)		56.94 (7.98)	56.48 (7.97)	56.61 (7.94)
No (%) consuming alcohol daily†	324 (25.1)	7544 (25.6)	46 342 (26.4)		256 (16.0)	5765 (16.5)	35 191 (16.90)
No (%) with haemochromatosis baseline diagnosis	156 (12.1)	56 (0.2)	55 (0.03)		54 (3.4)	17 (0.05)	16 (0.01)
Older ages (60-70 years):							
No (%)	593 (0.6)	13 599 (14.3)	80 945 (85.1)		719 (0.7)	14 905 (14.1)	90 214 (85.2)
Mean (SD) age (years)	64.19 (2.84)	64.29 (2.85)	64.25 (2.86)		64.26 (2.82)	64.02 (2.86)	64.04 (2.84)
No (%) consuming alcohol daily†	173 (29.2)	3962 (29.2)	24 277 (30.0)		125 (17.4)	2696 (18.1)	16 714 (18.5)
No (%) with haemochromatosis baseline diagnosis	81 (13.7)	31 (0.2)	35 (0.04)		34 (4.7)	7 (0.05)	5 (0.01)

* No p.C282Y mutations, irrespective of p.H63D status.

† 0.07% of participants with missing data were excluded.

Prevalent and incident diagnoses of haemochromatosis itself (irrespective of subtype) were also recorded for people with the p.C282Y heterozygote genotype, the p.H63D genotypes, and the p.C282Y/p.H63D composite genotype, with 10.7% of diagnoses recorded in participants with none of the studied mutations (see supplementary table 2 for prevalent diagnoses, and supplementary table 4 for incident diagnoses).

### p.C282Y homozygous status and prevalent conditions

**Fig 1:**
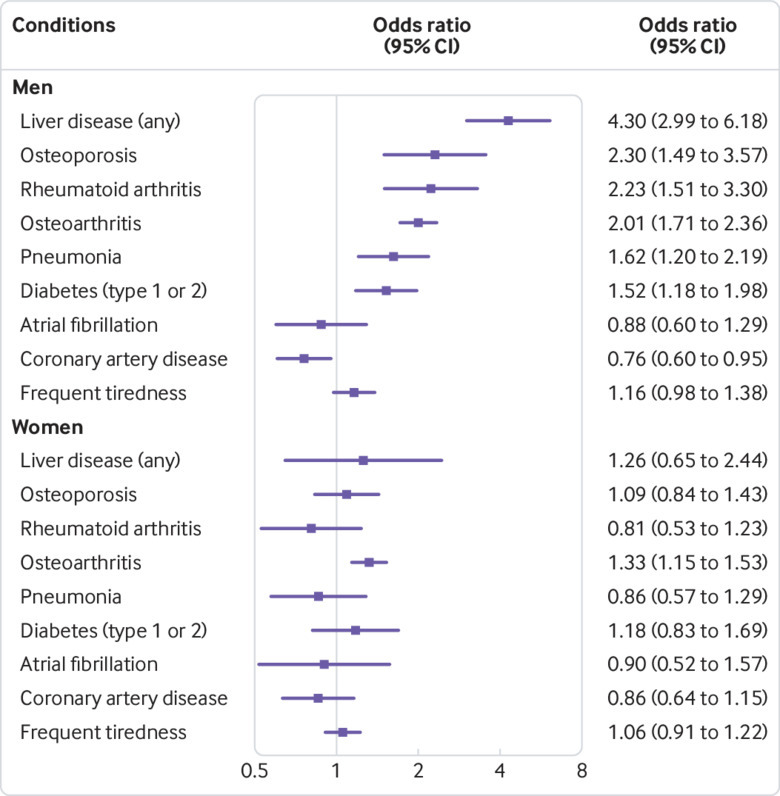
Prevalent conditions in studied sample (aged 40-70 at baseline): Forest plot of associations comparing p.C282Y homozygous status with no p.C282Y mutations, irrespective of p.H63D status, in men and women. Odds ratios are from logistic regression models adjusted for age, genotyping array type, and genetic principal components. See supplementary table 2 for details

Several associations with studied diagnoses were present in men (whole sample aged 40 to 70 years old at baseline, fig 1 and table 2; see supplementary table 2 for full results). Male p.C282Y homozygotes had a higher prevalence of diagnosed haemochromatosis (odds ratio 411.1, 95% confidence interval 299.0 to 565.3, P<0.001), as well as osteoarthritis (2.01, 1.71 to 2.36, P<0.001), liver disease (4.30, 2.97 to 6.18, P<0.001), rheumatoid arthritis (2.23, 1.51 to 3.31, P<0.001), and diabetes mellitus (1.53, 1.16 to 1.98, P=0.002), compared with no p.C282Y mutations (n=175 539, irrespective of H63D status). Associations were also present for osteoporosis (2.23, 1.49 to 3.57, P<0.001) and pneumonia (1.62, 1.20 to 2.19, P=0.002). Contrary to these increases in morbidity, diagnoses of coronary artery disease were less common (0.76, 0.61 to 0.95; P=0.016), although this association was no longer significant after correction for multiple testing (Benjamini-Hochberg corrected P=0.069): all other associations were still significant after multiple testing correction. No associations were found with tiredness or atrial fibrillations in men aged 40 to 70 years.

**Table 2:** Prevalence of p.C282Y associated baseline conditions by genotype (homozygotes, heterozygotes, and no pC282Y mutations, in participants aged 40-70 years. Values are numbers (percentages)

Baseline conditions	Men				Women		
	Homozygotes	Heterozygotes	No p.C282Y mutations*		Homozygotes	Heterozygotes	No p.C282Y mutations*
Haemochromatosis	156 (12.1)	56 (0.2)	55 (0.03)		54 (3.4)	17 (0.05)	16 (0.01)
Atrial fibrillation	27 (2.1)	769 (2.6)	4166 (2.4)		13 (0.8)	307 (0.9)	1751 (0.8)
CAD (myocardial infarction or angina)	84 (6.5)	2434 (8.2)	14 341 (8.2)		47 (2.9)	1141 (3.3)	6495 (3.1)
Diabetes (type 1 or 2)	61 (4.7)	1000 (3.4)	5523 (3.15)		31 (1.9)	568 (1.6)	3371 (1.6)
Liver disease	31 (2.4)	178 (0.6)	910 (0.5)		9 (0.6)	149 (0.4)	864 (0.4)
Osteoarthritis	182 (14.1)	2268 (7.7)	13 105 (7.5)		227 (14.2)	3780 (10.8)	22 522 (10.8)
Osteoporosis	21 (1.6)	206 (0.7)	1164 (0.7)		57 (3.57)	1042 (2.98)	6305 (3.03)
Pneumonia	45 (3.5)	654 (2.2)	3844 (2.2)		24 (1.5)	634 (1.8)	3626 (1.7)
Rheumatoid arthritis	26 (2.0)	249 (0.8)	1552 (0.9)		22 (1.4)	533 (1.5)	3399 (1.6)
Tiredness	68 (11.9)	1085 (8.3)	6455 (8.3)		76 (11.4)	1365 (9.65)	8630 (10.1)
≥1 core haemochromatosis diagnosis†	346 (26.7)	3518 (11.9)	19 778 (11.3)		304 (19.05)	4743 (13.6)	28 487 (13.7)
≥1 condition (any)	421 (32.5)	4922 (16.7)	28 143 (16.0)		398 (24.9)	6888 (19.7)	41 531 (19.9)

CAD=coronary artery disease.

* No p.C282Y mutations, irrespective of p.H63D status.

† Includes haemochromatosis; diabetes, liver disease, including liver cancer; osteoarthritis; and rheumatoid arthritis. Any associated condition also includes osteoporosis, pneumonia, and tiredness.

In women aged 40 to 70 years, p.C282Y homozygosity (n=1596, fig 1) was only associated with haemochromatosis (odds ratio 438.0, 95% confidence interval 247.9 to 773.9, P<0.001) and osteoarthritis (1.33, 1.15 to 1.53; P<0.001). Haemochromatosis was associated with p.C282Y homozygosity in all three 10 year age subgroups and in both sexes (see supplementary table 2). Most of the other conditions associated with p.C282Y homozygous status overall in men also reached statistical significance in the 50 to 59 years and 60 to 70 years age subgroups (see supplementary fig 1), whereas the association with osteoarthritis was present in all three age groups. Frequent tiredness or lethargy was associated with p.C282Y homozygosity in men aged 60 to 70 only (1.43, 1.10 to 1.84; P=0.007). In women, none of the age specific analyses reached significance.

We repeated prevalent analyses including an interaction term between p.C282Y genotype and sex, to test whether the difference seen between men and women was statistically significant. Sex significantly interacted with p.C282Y for prevalent osteoporosis (P=0.004 for interaction), osteoarthritis (P<0.001 for interaction), rheumatoid arthritis (P<0.001 for interaction), pneumonia (P=0.01 for interaction), and liver disease (P=0.001 for interaction). In all cases, being male increased the association.

### p.C282Y homozygous status associations with incident conditions

During follow-up (mean 7 (maximum 9.4) years), 107 p.C282Y homozygous participants died (hazard ratio 1.23, 95% confidence interval 1.01 to 1.48, P=0.04 compared with 11 092 deaths in participants with no p.C282Y mutations; see supplementary fig 2). Age group and sex specific associations with mortality did not reach significance, and the overall association with mortality was no longer significant after adjustment for multiple testing (Benjamini-Hochberg P=0.36; see supplementary table 3). Also see post hoc analyses of cause of death in the section on sensitivity analyses.

In men aged 40-70 years without a haemochromatosis diagnosis at baseline, p.C282Y homozygotes were more likely to have a diagnosis of haemochromatosis during follow-up compared with participants with no p.C282Y mutations (hazard ratio 286.2, 95% confidence interval 211.0 to 388.0). The p.C282Y homozygous women also had an increased likelihood of receiving a diagnosis of haemochromatosis (427.3, 285.4 to 639.7). Overall, male p.C282Y homozygotes were more likely to have a diagnosis during follow-up than female p.C282Y homozygotes (1.73, 1.33 to 2.25, P<0.001). Numbers of incident diagnoses in the p.C282Y homozygote group were relatively low (see supplementary table 4) but p.C282Y homozygote males aged 40 to 70 had a higher incidence of any liver disease (2.35, 1.60 to 3.43, P<0.001), as well as incident liver cancer (n=11 in p.C282Y homozygote males, hazard ratio 8.88, 95% confidence interval 4.79 to 16.45, P<0.001). Associations were also found with incident osteoarthritis in both men and women aged 40 to 70 (1.84, 1.20 to 2.80, P=0.005, and 1.81, 1.26 to 2.60, P=0.001, respectively) and with incident osteoporosis in the male p.C282Y homozygotes (2.18, 1.13 to 4.21, P=0.021), although this last association was no longer significant after correction for multiple testing (Benjamini-Hochberg P=0.14). We repeated analyses of incident diagnoses including an interaction term between sex and p.C282Y genotype: only liver cancer (P=0.038 for interaction) and liver disease (P=0.049 for interaction) interaction terms were statistically significant, with male sex increasing the estimated effect size.

**Fig 2:**
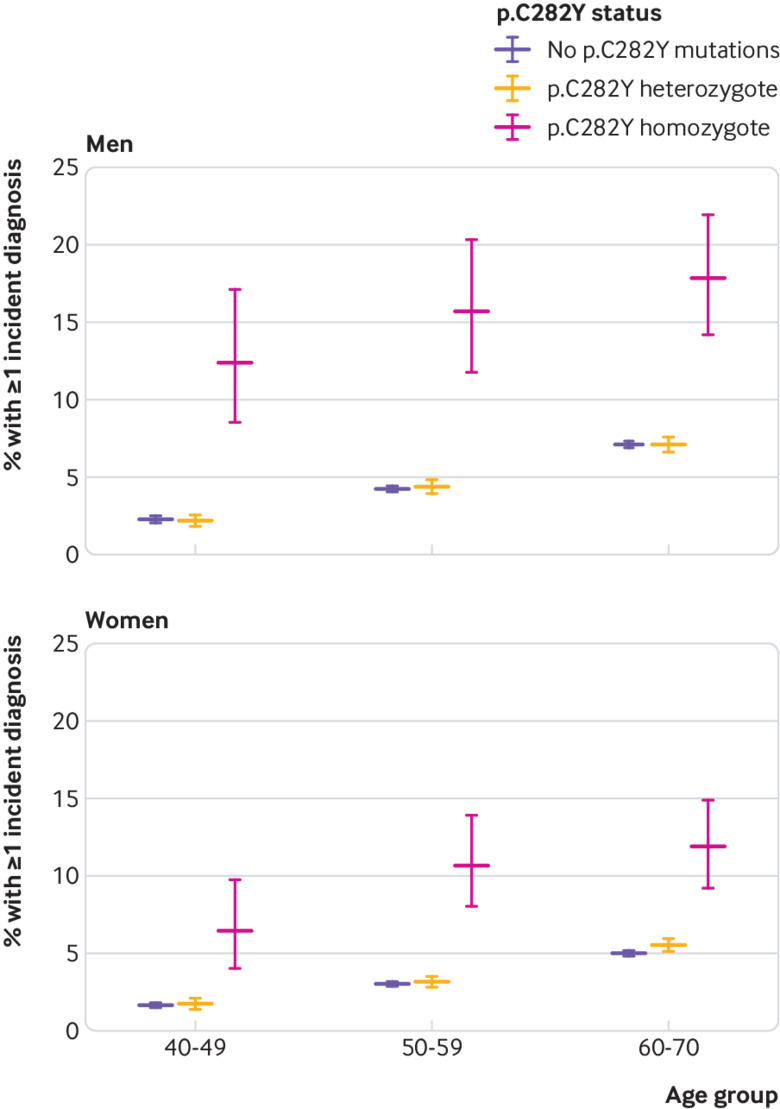
Percentage of participants with at least one incident diagnosis during follow-up, by age group, sex, and p.C282Y genotype. Absolute percentages and 95% confidence intervals are shown. Diagnoses included haemochromatosis, any liver disease, liver cancer, diabetes, osteoarthritis, and rheumatoid arthritis. Prevalent cases are excluded. All comparisons are with no p.C282Y mutations, irrespective of p.H63D status. See supplementary table 5 for details

**Fig 3:**
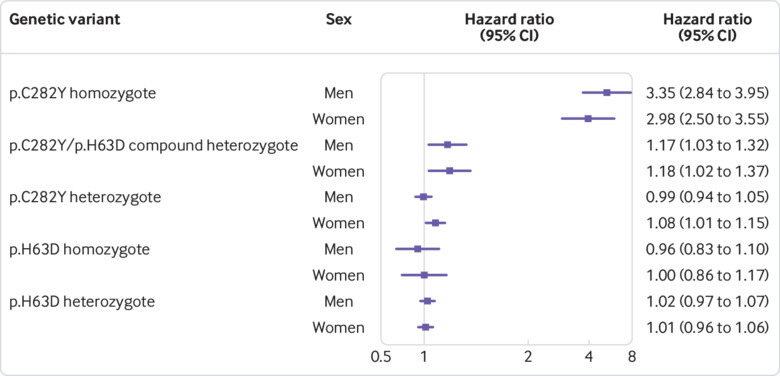
Forest plot of associations for developing at least one p.C282Y associated incident condition (incident haemochromatosis, liver disease (including liver cancer), diabetes (type 1 or 2), rheumatoid arthritis, or osteoarthritis) by end of follow-up, stratified by genotype and sex. Hazard ratios are from time-to-event (Cox’s proportional hazards regression) models adjusted for age, genotyping array type, and genetic principal components. The homozygous and heterozygous (p.C282Y/p.C282Y and p.C282Y/−, respectively) groups are compared with participants with no p.C282Y mutations, irrespective of p.H63D status. The p.C282Y/p.H63D group are compared with participants without p.C282Y and p.H63D mutations. p.H63D/p.H63D and p.H63D/− groups are compared with participants with no p.H63D mutations. See supplementary table 4 for details

As iron overload in haemochromatosis affects many parenchymal tissues, we also calculated a summary measure of excess incident morbidity, to allow estimation of overall absolute differences in morbidity. We estimated incidence of the core diagnoses linked to haemochromatosis—namely, haemochromatosis itself, any liver disease (including liver cancer), diabetes, osteoarthritis, or rheumatoid arthritis. During the seven year mean follow-up, 15.7% of the p.C282Y homozygote men (all ages) developed at least one “core” condition (after excluding prevalent cases) compared with 5.0% of men with no p.C282Y mutations (10.7 percentage point difference, 95% confidence interval 8.4 to 13.4; P<0.001); in women the respective estimates were 10.1% versus 3.4% (6.7 percentage point difference, 5.0 to 8.3; P<0.001, see supplementary table 5). These absolute estimates increase with age (fig 2, see supplementary table 5). In time-to-event analysis, both male and female p.C282Y homozygotes had higher hazards for incident morbidity (men: hazard ratio 3.37, 95% confidence interval 2.87 to 3.97, P<0.001; women 2.99, 2.51 to 3.55, P<0.001; fig 3; see supplementary figs 3 and 4). Sensitivity analyses combining the above diagnoses (haemochromatosis, any liver disease, liver cancer, diabetes, osteoarthritis, and rheumatoid arthritis) with osteoporosis and pneumonia gave slightly higher estimates: 19.3% of p.C282Y male homozygotes aged 40-70 years developed at least one condition during follow-up (after excluding prevalent cases) compared with 8.7% of participants with no p.C282Y mutations (10.6% difference, 95% confidence interval 13.2% to 8.1%, P<0.001). In women, the respective estimates were 12.4% versus 6.2% (6.2% difference, 8.0% to 4.4%, P<0.001).

### Overall penetrance in p.C282Y homozygotes

Overall penetrance (ie, having at least one diagnosis of haemochromatosis; any liver disease, including liver cancer; diabetes; osteoarthritis; or rheumatoid arthritis, including prevalent and incident diagnoses at end of follow-up, mean age 63.8 years) was 38.3% in p.C282Y homozygous men (all ages) compared with 15.7% in men with no p.C282Y mutations—that is, an additional 22.6% of male p.C282Y homozygotes developed one or more of these diagnoses (95% confidence interval 19.9% to 25.2%, P<0.001, with the highest absolute rates in the older groups. In women, the respective estimates were 27.2% (25.0% to 29.4%) versus 16.6% (16.6% to 16.8%), excess morbidity 10.6% (8.4% to 12.8%, P<0.001) (see supplementary fig 5 and supplementary table 5).

### Incident morbidity in p.C282Y/p.H63D compound heterozygotes

Of the 451 243 participants, 10 701 (2.4%) were p.C282Y/p.H63D compound heterozygotes (4955 were men, 46.3%). Mortality was not increased in p.C282Y/p.H63D compound heterozygotes (P=0.31) compared with participants with neither p.C282Y nor p.H63D mutations.

After excluding 79 participants with a prevalent diagnosis of haemochromatosis, compound heterozygotes had an increased likelihood of incident haemochromatosis diagnoses (men n=46, compound heterozygote cases, hazard ratio 33.63, 95% confidence interval 21.44 to 52.76, P<0.001; women n=17, 34.74, 16.47 to 73.29, P<0.001) compared with participants with neither p.C282Y nor p.H63D mutations (n=268 577, of which 122 841 were men, 45.7%).

The compound heterozygous participants were also more likely to have at least one incident diagnosis of haemochromatosis; any liver disease, including liver cancer; diabetes; osteoarthritis; or rheumatoid arthritis (men: hazard ratio 1.17, 95% confidence interval 1.03 to 1.33, P=0.016; women: 1.18, 1.03 to 1.37, P=0.022; fig 3; see supplementary table 4 for details), although these associations were no longer significant after correction for multiple testing (Benjamini-Hochberg P=0.14 and 0.14, respectively).

### Incident morbidity in p.C282Y heterozygotes

Overall, 64 444 participants were heterozygous for p.C282Y (irrespective of p.H63D status, 14.3%), of whom 29 526 were men (45.8% of 64 444). Mortality was not increased in p.C282Y heterozygotes (P>0.05) compared with participants with no p.C282Y mutations. After excluding participants with prevalent haemochromatosis, the p.C282Y heterozygotes had an increased likelihood of a haemochromatosis diagnosis (men: hazard ratio 5.77, 95% confidence interval 4.09 to 8.13, P<0.001; women: 5.30, 3.201 to 8.78, P<0.001) compared with no p.C282Y mutations (n=383 909 (45.72%) of whom 175 539 were men).

After excluding 10 660 p.C282Y/p.H63D compound heterozygotes, simple p.C282Y heterozygotes only were still more likely to have a diagnosis of incident haemochromatosis (3.42, 2.17 to 5.38, P<0.001) compared with those with no p.C282Y or p.H63D mutations. We observed no excess morbidity (at least one diagnosis of incident haemochromatosis; any liver disease, including liver cancer; diabetes; osteoarthritis; or rheumatoid arthritis) in the p.C282Y heterozygous men (P=0.78) and a nominally increased likelihood in the p.C282Y heterozygous women (1.08, 1.01 to 1.15, P=0.023) compared with those with no p.C282Y mutations (fig 3). After excluding 10 660 p.C282Y/p.H63D compound heterozygotes, p.C282Y heterozygous women no longer had an increased risk of incident excess morbidity (1.07, 0.99 to 1.15, P=0.07) compared with women with no p.C282Y or p.H63D mutations.

### Interactions with small effect iron variants

We tested for interactions between p.C282Y and a single genetic risk score for increased iron levels in men aged 40-70 (combining three alleles associated with iron levels,^19^ weighted for effect size, excluding p.C282Y and p.H63D: see supplementary information). We found no significant (P>0.05) interactions for the prevalent or incident diseases individually (see supplementary table 6). However, for incident excess morbidity (at least one diagnosis of haemochromatosis; any liver disease, including liver cancer; diabetes; osteoarthritis; or rheumatoid arthritis) a statistical interaction between p.C282Y homozygosity and the iron single genetic risk score (P=0.001, Benjamini-Hochberg P=0.018) was present. In further investigations, p.C282Y homozygous men above the mean iron single genetic risk score had a 51% increased risk of an incident diagnosis (of haemochromatosis; any liver disease, including liver cancer; diabetes; osteoarthritis; or rheumatoid arthritis; hazard ratio 1.51, 95% confidence interval 1.09 to 2.09, P=0.01) compared with p.C282Y homozygotes below the mean iron single genetic risk score.

### Sensitivity analyses

Given the associations of p.C282Y homozygosity with lower prevalence of coronary artery disease in men aged 40 to 70 at baseline, we tested associations with self reported use of cholesterol lowering drugs (47 141 of 206 175 men (22.9%)). p.C282Y homozygous men (aged 40 to 70) had a reduced likelihood of using cholesterol lowering drugs at baseline (odds ratio 0.76, 95% confidence interval 0.68 to 0.85; P<0.001) compared with men with no p.C282Y mutations. p.C282Y homozygous women (aged 40-70) also reported less use of cholesterol lowering drugs (0.75, 0.67 to 0.84; P<0.001), although the p.C282Y association with coronary artery disease was not significant in this group (P>0.05). We also repeated the analyses of the significant associations of p.C282Y with prevalent and incident diagnoses excluding participants self reporting use of cholesterol lowering drugs. All associations remained significant, with consistent or greater effect estimates (except for prevalent coronary artery disease, which became non-significant after removing the treated cardiovascular disease risk group: P=0.8) (see supplementary table 7 for full results). Given the p.C282Y homozygote associations with arthritis and osteoporosis, we undertook additional analyses on self reported receipt of hip replacement at baseline (to provide a hard endpoint): both male and female p.C282Y homozygotes had higher rates of hip replacement (2.62, 1.97 to 3.49, P<0.001 and 1.90, 1.52 to 2.54, P<0.001, respectively) compared with participants with no p.C282Y mutations.

Given the nominally significant p.C282Y homozygote association with excess mortality (when combining men and women, aged 40 to 70 at baseline) during follow-up, we undertook post hoc analyses on causes of death. The doctor recorded primary cause for 14 of these deaths was liver cancer (hepatic cell carcinoma n=10, intrahepatic bile duct carcinoma n=4), and 12 of these liver cancers occurred in male p.C282Y homozygotes. The odds ratio for p.C282Y homozygotes for liver cancer as a cause of death was 8.40 (95% confidence interval 4.68 to 15.05, P<0.001) compared with no p.C282Y mutations: men 14.01 (7.20 to 27.28, P<0.001); not significant in females P=0.26. No other main underlying causes of death were recorded in more than 10 of the p.C282Y homozygous participants who died.

The UK Biobank sample studied included 27 573 pairs of first degree relatives (KING kinship coefficient >0.177). To avoid inflation of associations from family effects, we excluded one randomly selected participant from each pair related to the third degree (kinship coefficient >0.0442) or closer (n=71 651), leaving 379 592 unrelated participants for sensitivity analyses. The results were similar to the main results (see supplementary table 8): the proportion of male p.C282Y homozygotes aged 40 to 70 with at least one positively associated diagnosis (haemochromatosis; any liver disease, including liver cancer; diabetes; rheumatoid arthritis; or osteoarthritis) was 14.6%, compared with 5.0% in those with no p.C282Y mutations (absolute excess 9.58 percentage points, 95% confidence interval 7.12 to 12.05): the comparative results for the overall sample were 15.7% and 5.0%, respectively, suggesting that inclusion or exclusion has little effect on estimates.

We tested for multiplicative risk effects (statistical interaction terms) between p.C282Y homozygous status and daily alcohol consumption in men aged 40 to 70 for each baseline associated condition, plus the combined measure of morbidity: no interactions were significant (P>0.05), implying that daily alcohol intake seems to be an additive risk factor for studied outcomes. In further analyses, 88.4% of men aged 40-70 self reported weekly alcohol intake, and those in the top 20% of alcohol intake consumed more than 37.2 units (1 unit=10 mL) each week. In men with no p.C282Y mutations, being in the top 20% of alcohol intake was associated with an increased risk of incident liver disease (hazard ratio 1.75, 95% confidence interval 1.55 to 1.96, P<0.001) compared with being in the other alcohol intake groups (excluding those with missing data). Similarly, male p.C282Y homozygotes in the top 20% of alcohol intake also had raised hazards for incident liver disease (2.49, 1.02 to 6.06, P=0.04), but confidence intervals were wide and overlapped with the previous estimate.

We repeated the primary analyses of incident diagnoses using competing risks regression models, with all cause mortality as the competing risk.^22^ We found that including mortality as a competing risk altered the associations only slightly (see supplementary table 9), suggesting that the primary results presented from Cox’s proportional hazards regression models are reliable and not biased by including participants who died in the censored group.

We investigated whether the *HFE* p.C282Y associations with specific diseases could be due to statistical co-localisation with other genetic variants in the region (see supplementary methods). No strong evidence supported a likely causal role for other variants, although five of the seven potential candidates were not directly genotyped, so further work may be justified (see supplementary results).

As measures of transferrin saturation were not available, we modelled transferrin saturation related genetic variant associations with diagnoses using mendelian randomisation approaches (supplementary tables 10 and 11). In men, increased genetically instrumented transferrin saturation (reported by Allen et al^23^) was associated with incident haemochromatosis (inverse variance weighted β=0.15, P<0.001), liver disease (β=0.016, P=0.005), liver cancer (β=0.047, P<0.001), osteoarthritis (β=0.012, P=0.005), osteoporosis (β=0.015, P=0.02), but not diabetes (type 1 or 2: β=0.006, P=0.06). In women, increased genetically instrumented transferrin saturation was associated with incident haemochromatosis (β=0.25, P<0.001) and osteoarthritis (β=0.015, P=0.002). The associations were consistent in the sensitivity analyses. Full results are available in supplementary table 11, and supplementary figures 8-14 show the individual *HFE* genotypes plotted against incident disease risk. Caution is needed in interpreting these results as the methods might not apply to the extreme transferrin saturation levels seen in *HFE* genotypes (eg, Allen et al^23^ reported a mean transferrin saturation of 73% in male p.C282Y homozygotes compared with 30% in males with no p.C282Y mutations; that is, more than 40 standard deviations above the mean for the wild type genotype).

Finally, to further explore relevance to daily clinical practice in the UK, we estimated the proportions of cases of each condition that occurred in p.C282Y homozygous men and women. In men, 1.59% of all hip replacements at baseline were in p.C282Y homozygotes. During follow-up 5.82% of all incident liver cancers in the studied UK Biobank sample occurred in the p.C282Y homozygotes (see supplementary table 4), 1.45% of incident liver diseases, 1.40% of incident osteoporosis, 1.12% of incident osteoarthritis, 0.89% of incident diabetes (type 1 or 2), 0.86% of incident rheumatoid arthritis, and 0.67% of incident pneumonia in the studied UK Biobank sample occurred in male p.C282Y homozygotes.

## Discussion

Previous evidence suggested that penetrance to disease in the highest risk hereditary haemochromatosis *HFE* p.C282Y homozygote family members was common, but penetrance in community samples was reportedly low. We studied a large UK based community volunteer sample aged 40 to 70 at baseline, with a mean seven year follow-up. p.C282Y homozygous men aged 40 to 70 had substantial excess morbidity at baseline across several body systems typically affected in haemochromatosis, with increased prevalence of haemochromatosis itself, diabetes, rheumatoid arthritis, osteoarthritis, and liver disease compared with people with no p.C282Y mutations. In prospective analyses during follow-up, p.C282Y homozygous status was also associated with substantial incident morbidity in both men and women: 15.7% of the men developed at least one incident haemochromatosis, diabetes, arthritis, or liver disease compared with 5.0% in men with no p.C282Y mutations, with the respective estimates for women being 10.1% and 3.4%. These incidence rates alone indicate penetrance to diagnosed disease in p.C282Y homozygotes was not uncommon in our community sample. We also found a nominally significant increase in mortality in the p.C282Y homozygotes overall (despite reduced prevalence of coronary artery disease in men), although numbers of deaths were small. Excess morbidity with the other studied genotypes (p.C282Y heterozygote, p.C282Y/p.H63D composite genotype) was modest, although these genotypes were associated with excess diagnoses of haemochromatosis.

### Limitations and comparisons with previous studies

Although the UK Biobank is a volunteer sample, our observed prevalence of p.C282Y heterozygote status (14.3% in UK Biobank) is similar to the 14.1% in a group of predominantly British or Irish descent in Australia.^24^ Our results are broadly consistent with those of Asberg et al,^25^ who studied transferrin saturation levels in 65 238 Norwegians (mean age 49 years) in a population based health survey and found p.C282Y homozygous status in 171 men (population prevalence 0.68%) and 126 women (prevalence 0.41%) with serological evidence of iron overload. Overall, 57% of these men and 52% of these p.C282Y homozygote women had raised alanine aminotransferase concentrations (suggesting liver involvement), plus fatigue, joint pain, impotence (men), or diabetes mellitus, with joint pain alone being present in 20.3% of the men and 13.0% of the women. Other large sample population representative data on p.C282Y health outcomes are limited, with the largest study (HEIRS^9^) examining genotypes in 99 711 North American participants from primary care practices and blood sampling laboratories (median age 50, n=299 p.C282Y homozygotes). HEIRS found only an association between p.C282Y homozygous status and any liver disease in men. Beutler et al^9^ studied 41 038 individuals who had chosen to attend health appraisal clinics offered by Kaiser Permanente in California, with the 152 p.C282Y homozygotes having only excess “liver trouble” or hepatitis, and the authors concluded a “best estimate” that less than 1% of homozygotes develop “frank clinical haemochromatosis.” Even the largest previous studies contained relatively small numbers of p.C282Y homozygote individuals compared with UK Biobank (n=2890 versus HEIRS^9^ n=299, Kaiser Permanente n=152), limiting their statistical power to detect excess diagnoses: for example, Beutler et al^10^ had only 19% power at P=0.05 to detect our p.C282Y male homozygote association with diabetes (odds ratio 1.44 in UK Biobank) in their sample of 56 p.C282Y homozygous men. In the UK Biobank analysis of incident diabetes, we had 80% power to detect an odds ratio of 1.034 in our sample of 1281 p.C282Y homozygous men compared with 174 760 controls compared to controls with no p.C282Y mutations (n=6358 incident cases versus 169 683 controls), with P<0.05.

Many smaller studies^26^ examined p.C282Y associations using varying methodology and with varying results, with overall clinical penetrance being low in the predominantly p.C282Y heterozygotes studied. We also found a low incidence of haemochromatosis associated diagnoses with p.C282Y heterozygote status alone (compared with no p.C282Y mutations, excluding p.C282Y homozygotes). We did find one modest association between the compound heterozygous p.C282Y/p.H63D genotype and excess incident morbidity in men aged 40 to 49. More work is needed to establish whether this association replicates in other populations and whether interacting factors increase the risk of disease in this and the other genotypes. Our results included a nominally significant association with mortality in the combined male and female p.C282Y homozygote group, although this was based on only 107 deaths: previous studies have reported increased mortality in patients with hereditary haemochromatosis and liver disease, but not otherwise.^27^ ^28^ Liver cancer was recorded on the death certificates of 14 of the 107 p.C282Y homozygous decedents in our study. In analyses of specific incident diagnoses we have accounted for this differential mortality using competing risks analyses in a sensitivity analysis: this made little difference to the results, likely because of the relatively small numbers of deaths.

One beneficial aspect of p.C282Y homozygous status is our finding that prevalence of coronary artery disease was reduced in men, as was use of cholesterol lowering drugs in both men and women aged 40-70. This is consistent with the lower low density lipoprotein cholesterol levels previously reported^7^ ^28^ in p.C282Y homozygotes. Miller and Hutchins^29^ found a reduced prevalence of coronary artery disease in patients with haemochromatosis. In a mendelian randomisation analysis, Gill et al^30^ recently found that higher genetically determined iron levels were associated with reduced risk of coronary artery disease (although separate data on *HFE* p.C282Y homozygotes were not modelled). Further application of mendelian randomisation analysis could help clarify the contribution of iron levels to other studied morbidities.

Other limitations of our analyses include that UK Biobank volunteers tended to be healthier than the general population^21^ at baseline. Also, it is possible that response rates to UK biobank may have been affected by *HFE* mutation status or associated morbidity. As noted, the overall prevalence of p.C282Y homozygosity (1 in 156) was similar to previous reports for groups of British or Irish descent, and the p.C282Y variant was in Hardy-Weinberg equilibrium (P>0.05) in UK Biobank, implying that the observed genotypes are present in the expected proportions, with no sign of differential loss or excess of p.C282Y homozygotes. More importantly, we found high rates of incident haemochromatosis associated diagnoses with p.C282Y homozygosity during the follow-up period, after excluding people with prevalent diagnoses at baseline. The UK Biobank sample included a wide range of exposures and socioeconomically diverse groups,^21^ and prospective analyses are much less affected by sample response patterns at baseline. These factors and the similarity of our findings to the Norwegian population sample by Asberg et al^25^ suggest that our results are robust and likely to be applicable to the UK population and other populations of European descent.

The UK Biobank sample included some sets of related people, as assessed through genome wide variant similarity (KING kinship coefficient). In our incident estimates, we excluded those with existing haemochromatosis diagnoses at baseline and found that results were similar whether we included or excluded up to third degree relatives (see sensitivity analyses and supplementary table 6). However, UK Biobank lacks data on whether each related or unrelated UK Biobank participant was from a family with a strong history of haemochromatosis diagnoses.

For incident diagnoses, we used competing risk regression models accounting for the competing risk of mortality in sensitivity analyses, as many people only receive a diagnosis of haemochromatosis at older ages, when background mortality is substantial (see for example the eMERGE^31^ clinical biobank study across seven North American medical centres), although estimates using simple Cox regression yielded similar results (see supplementary information). We used proportional hazards regression models for incidence estimates, which assume that hazards do not vary over time. Tests of this assumption were negative for all outcomes tested, with one exception: diabetes in men, which showed few cases in the p.C282Y homozygotes during the first two years (see supplementary information). After excluding the first two years of data, the association between p.C282Y homozygosity and incident diabetes no longer had a significant violation of the proportional hazards assumption. Overall therefore we found no evidence of excess morbidity in p.C282Y homozygotes in the early years of follow-up, as might happen if these people were more likely to take part in this volunteer genetics study because of pre-existing morbidity.

Our analysis lacks measures of iron overload. Serum ferritin concentrations are reportedly increased in 80% of men (>300 µg/L) and 50% of women (>200 µg/L) with p.C282Y homozygosity.^32^ In an Australian population cohort of median age 65 years, serological evidence of iron overload (serum ferritin ≥1000 μg/L, considered serious by the original authors) was found in 35% of male and 6% of female p.C282Y homozygotes.^23^ In a study of genotyped patients in eMERGE clinical biobanks across seven US health systems,^31^ 100% of the male and 50% of the female p.C282Y homozygotes had transferrin saturations above 50%. Higher levels of iron deposition with *HFE* genotypes were also reported in preliminary findings from UK Biobank subgroups for both liver imaging (with seven of eight studied p.C282Y homozygotes having evidence of iron overload^33^) and with *HFE* genotype for iron deposition in certain brain regions.^34^ Future work including serum iron related measures could help identify the factors that influence why some p.C282Y homozygotes develop associated disease but not others and might also help inform translational studies to improve diagnosis.

We have been able to study mainly clinical diagnoses, but misdiagnoses of iron related disease have been reported—for example, with the iron deposition related arthrosis misdiagnosed as osteoarthritis or rheumatoid arthritis.^35^ However, our results for p.C282Y homozygote associations with musculoskeletal diagnoses^36^ (including^16^ ^17^ ^37^ ^38^ arthritis, hip replacement, and osteoporosis) are consistent with previous reports. As follow-up is currently limited in duration and does not include primary care recorded diagnoses, our estimates of excess morbidity are likely to be conservative.

We have avoided adjustment for commonly examined confounders in observational studies, such as smoking, diet, or exercise, as the genotypes studied are inherited at conception and are not altered by later exposure. As p.C282Y homozygote men and women had more arthritis at baseline, it would not be surprising if they therefore did less physical exercise, and adjustment for such downstream effects could bias results. Instead we have provided sensitivity analyses on alcohol intake, as the main previously studied modifying exposure.

Adjustment for multiple statistical testing is particularly important when examining many statistical associations for unrelated outcomes, and with low prior probabilities of associations being present. However, our analysis is essentially a replication of previous similar studies. The deposition of excess iron in hereditary haemochromatosis results in widespread damage to parenchymal tissue, leading to several different diagnoses through the common mechanism of iron accumulation.^15^ It is also established that incidence of haemochromatosis is higher in men than in women, and that it increases with advancing age. There is extensive evidence for associations of the studied comparisons in patients with haemochromatosis or with the p.C282Y genotype, or both, so prior probabilities were high, although unquantified in a large community sample. To further deal with multiple testing we selected only ascertainable conditions previously linked to haemochromatosis, clearly identified the post hoc sensitivity analyses, and provided a summary estimate of all excess incident diagnoses together. We have also reported Benjamini-Hochberg adjusted P values to quantify multiple testing adjusted significance: more data are needed especially on associations that were no longer significant.

### Clinical and policy implications

Previous community level studies of people homozygous for p.C282Y have suggested low penetrance, with Beutler et al^10^ estimating that less than 1% develop “frank clinical haemochromatosis.” In UK Biobank, haemochromatosis was eventually diagnosed in 21.7% of male p.C282Y homozygotes and 9.8% of women p.C282Y homozygotes, by end of follow-up. These diagnostic rates are comparable to the eMERGE^31^ clinical biobank study across seven North American medical centres, which found that overall 24.4% of men and 14.0% of women p.C282Y homozygotes had a diagnosis of haemochromatosis (n=106, mean age of diagnosis 61.5 years). Interestingly, survival curves to diagnosis in eMERGE suggested that by age 90, nearly 50% of the men and approximately 25% of the women p.C282Y homozygotes had a diagnosis of haemochromatosis. It is clear therefore that clinically diagnosed haemochromatosis eventually develops in large proportions of p.C282Y homozygotes.

Phlebotomy and other iron reducing interventions are effective at improving clinical outcomes^5^ in individuals with high iron levels, especially when started before development of the related pathologies: after pathologies are established, effectiveness is limited, especially for arthrosis. In addition, a recent randomised trial^39^ using erythrocytapheresis in p.C282Y homozygotes with moderately increased serum ferritin concentrations reported improvements in patient reported fatigue, with few adverse events. It seems likely that intervention before the development of pathologies in p.C282Y homozygotes in the community could prevent a substantial burden of excess morbidity.

### Future work

More work is needed to confirm p.C282Y homozygous associations with mortality over longer follow-ups, with further elucidation of causes of death. Work is also needed to establish whether even greater excess morbidity develops at older ages than studied here, especially in women, as time since menopause increases. Large scale studies of p.C282Y homozygotes including direct measures of iron status could also help identify additional risk factors and improve prediction of outcomes. Further data on morbidity associated with the lower risk *HFE* variants is also needed. Further analyses are needed to identify possible additional variants contributing to haemochromatosis penetrance, and further analysis of statistical co-localisation could help disentangle shared and distinct causal variants for each of the various clinical manifestations of haemochromatosis.

More work is needed on whether low density lipoprotein cholesterol levels increase after iron reduction intervention, and whether the risk of coronary artery disease can be kept low with statins and other preventive interventions. It might be that prevention of coronary artery disease could replace the beneficial effect of higher iron levels on coronary artery disease outcomes in p.C282Y homozygotes, unmasking the full detrimental effects of iron accumulation on survival.

Although hereditary haemochromatosis is regarded as fitting several criteria for genetic screening,^10^ the previously reported low clinical penetrance in community p.C282Y carriers did not justify screening beyond close relatives. In the light of the accumulating evidence that penetrance to clinically diagnosed morbidity in p.C282Y homozygotes is not uncommon and that many cases are missed or diagnosed only after substantial morbidity has developed, more work is needed to evaluate the various possible approaches to improve prevention and early case ascertainment. Issues to evaluate include the various options for testing (eg, with initial serum ferritin or transferrin saturation assays, or perhaps through initial genotyping), and the different population or patient groups who might be targeted.

### Conclusions

In the large UK Biobank community sample, *HFE* p.C282Y homozygotes experienced substantial excess prevalent and incident clinical morbidity. This excess morbidity was most evident in men but was also present in women, and it was more common with advancing age. As p.C282Y associated iron overload is preventable and partly treatable, re-examination of the many issues involved in recommending expanded early case ascertainment or screening is justified.

**What is already known on this topic**

The *HFE* p.C282Y homozygous mutation is the main cause of the iron overload disorder hereditary haemochromatosis (type 1) in populations of European descent
Iron overload is preventable and partly treatable by venesection, but diagnosis is often missed or delayed
Community studies reported low penetrance, with one reporting that less than 1% of p.C282Y homozygotes developed frank clinical haemochromatosis

**What this study adds**

Combining baseline and incident diagnoses (mean age 63.8), 1 in 5 more male p.C282Y homozygotes were diagnosed as having one or more of haemochromatosis, any liver disease, diabetes mellitus, rheumatoid arthritis, or osteoarthritis, compared with those without mutations (ignoring p.H63D)
In women, 1 in 10 more developed morbidity
Issues involved in offering screening, and improving early case ascertainment for p.C282Y homozygotes need re-examining

## Web extra

Extra material supplied by authors

**Supplementary information:** additional methods, results, and figures, including a link to additional tables

## Notes

Contributors: LCP performed the analysis, interpreted results, created the figures, searched literature, and cowrote the manuscript. JT performed the literature search, performed analyses, and contributed to the manuscript. GJ performed analyses and contributed to the manuscript. AW performed analyses relating to the genetic classification of participant ancestry. LJ, CK, GAK, and LF contributed to data interpretation and contributed to the manuscript. DM oversaw data analysis and interpretation and literature searching and led the writing of the manuscript. DM is the guarantor. The corresponding author attests that all listed authors meet authorship criteria and that no others meeting the criteria have been omitted.

Ethical approval: The North West Multi-Centre Research Ethics Committee approved the collection and use of UK Biobank data.

Data sharing: Data are available on application to the UK Biobank (www.ukbiobank.ac.uk/register-apply).

Transparency: The lead author (DM) affirms that this manuscript is an honest, accurate, and transparent account of the study being reported; that no important aspects of the study have been omitted; and that any discrepancies from the study as planned (and, if relevant, registered) have been explained.
