# Supplementary material for: Common conditions associated with hereditary haemochromatosis genetic variants: cohort study in UK Biobank
Source: BMJ. 2019 Jan 16;364:k5222. doi: 10.1136/bmj.k5222 (PMC6334179; doi:10.1136/bmj.k5222)
Supplement: Supplementary file 1 — Supplementary information: additional methods, results, and figures, including a link to additional tables [file pill044661.ww1.pdf]

Common conditions associated with hereditary haemochromatosis genetic variants: cohort study in UK Biobank

Pilling *et al.*
BMJ 2019;364:k5222 doi: 10.1136/bmj.k5222

Supplementary Information

Supplementary Methods 3
Genotyping 3
Disease ascertainment 3
Proportional hazards assumption 3
Statistical Colocalisation 4
Supplementary Results 5
Proportional hazards assumption 5
Comparing Cox's proportional hazards to competing risks regression 5
Statistical Colocalisation 5
Supplementary Figures 6
Supplementary Figure 1 – Prevalent conditions by age-subgroup and sex: Forest plot of associations (Odds Ratios) comparing p.C282Y homozygous status to wild type (no p.C282Y) in A) males and B) females separately. 6
Supplementary Figure 2 – survival curve for p.C282Y genotypes and mortality 7
Supplementary Figure 3 – survival curve for p.C282Y genotype and any incident diagnosis (males) 8
Supplementary Figure 4 – survival curve for p.C282Y genotype and any incident diagnosis (females) 8
Supplementary Figure 5 – overall excess incident morbidity 9
Supplementary Figure 6 – survival curve for p.C282Y genotype and incident diabetes (males) 10
Supplementary Figure 7 – survival curve for p.C282Y genotype and incident diabetes (males) excluding first 2 years 10
Supplementary Figure 8 – MR analysis of transferrin saturation and haemochromatosis in males 11
Supplementary Figure 9 – MR analysis of transferrin saturation and liver disease in males 11
Supplementary Figure 10 – MR analysis of transferrin saturation and liver cancer in males 12
Supplementary Figure 11 – MR analysis of transferrin saturation and osteoarthritis in males 12
Supplementary Figure 12 – MR analysis of transferrin saturation and osteoporosis in males 13
Supplementary Figure 13 – MR analysis of transferrin saturation and haemochromatosis in females 14
Supplementary Figure 14 – MR analysis of transferrin saturation and osteoarthritis in females 14
Supplementary Figure 15 – Colocalisation analysis of prevalent haemochromatosis in males 15
Supplementary Figure 16 – Colocalisation analysis of prevalent coronary heart disease (CHD) in males 16
Supplementary Figure 17 – Colocalisation analysis of prevalent diabetes (type 1 or 2) in males 17
Supplementary Figure 18 – Colocalisation analysis of prevalent liver disease in males 18
Supplementary Figure 19 – Colocalisation analysis of prevalent osteoarthritis in males 19

Supplementary Figure 20 – Colocalisation analysis of prevalent osteoporosis in males 20
Supplementary Figure 21 – Colocalisation analysis of prevalent pneumonia in males.................................. 21
Supplementary Figure 22 – Colocalisation analysis of prevalent Rheumatoid arthritis in males 22
Supplementary Figure 23 – Colocalisation analysis of ≥1 associated prevalent diagnosis in males................ 23
Supplementary Figure 24 – Colocalisation analysis of prevalent haemochromatosis in females 24
Supplementary Figure 25 – Colocalisation analysis of prevalent osteoarthritis in females 25
Supplementary Figure 26 – Colocalisation analysis of ≥1 prevalent diagnosis in females 26

References 27

Supplementary Tables available to download separately:

http://dx.doi.org/10.6084/m9.figshare.7371179

Supplementary Methods

Genotyping

Genetic data were available on 488,377 UK Biobank participants after genotype calling and quality control performed centrally by the UK Biobank team.[1] In this analysis we used data from the UK Biobank v2 imputed genotype release (2017). Imputation of genetic variants from the Haplotype Reference Consortium panel was performed using IMPUTE4 centrally by the UK Biobank team [1]; 487,409 subjects genotypes were successfully imputed to the Haplotype Reference Consortium (HRC) (genotype imputation did not change between the v2 and v3 release for the analysed variants). We selected 451,427 participants identified as 'white European' through self-report and verified through principal components (PC) analysis based on genotypes. Briefly, PCs were generated in the 1000 Genomes Cohort using high-confidence genetic variants to obtain their individual loadings. These loadings were used to project UK Biobank participants into the same PC space. PCs 1 to 4 were used to identify participants of European descent Related individuals were identified through kinship analysis.

p.C282Y is a single nucleotide polymorphism (SNP) on chromosome 6 (b37 position 26093141, rsID rs1800562) in the *HFE* gene. H63D is a SNP also in the *HFE* gene (b37 position 26091179, rsID rs1799945). Both are missense variants causing a single amino-acid change in HFE. In the UK Biobank participants the SNPs were not correlated (R2=0.015). Three other SNPs outside the *HFE* gene (rs8177240, rs7385804, and rs855791) were used in this analysis, as they are known to affect circulating iron levels[2]. rs1800562 was not directly genotyped, therefore imputed p.C282Y genotypes were used (as previously mentioned, the v2 data from UK Biobank was used for analyses, but the data for this variant is identical in the 2018 v3 release): 445,521 participants (98.7% of 451,427) were imputed with 100% confidence and 5,723 were recoded (i.e. estimated genotype dose between 0 and 0.25 set to 0, values between 0.75 and 1.25 set to 1, and finally between 1.75 and 2 to 2); 183 participants (0.04%) were excluded due to imprecise imputation, yielding 451,243 participants in analyses.

Disease ascertainment

Disease ascertainment was by self-reported doctor diagnoses at baseline, plus diagnoses recorded in linked ICD10 coded inpatient hospital records covering the period 1996 to the date of participant baseline interview. Principal outcomes were those reportedly associated with hemochromatosis, plus related measures and common conditions in older groups. See supplementary table 1 for details.

Self-reported frequency of tiredness or lethargy in last 2 weeks at UKB baseline (field ID=2080) was reclassified from responses "not at all", "several days", "more than half the days" and "nearly every day", combining the latter two categories, compared to the rest. Participants who responded "do not know" or "prefer not to answer" were excluded from analyses (<3% of responders). Cholesterol lowering medication field IDs = 6153 and 6177. Alcohol field ID = 1558.

Incident hospital admissions data were from Hospital Episode Statistics (HES) for England, the Patient Episode Database for Wales (PEDW) and the Scottish Morbidity Record (SMR) [3]. Incident cancer registrations were from the National Health Service Information Centre (NHS IC) for England and Wales, National Records of Scotland and NHS Central Register. National death certification was also used.

Proportional hazards assumption

An assumption of Cox's proportional hazards (and competing risks) regression models is that the hazard ratio remains constant over time. We used the STATA function `stphtest` to test whether the proportional hazards assumption was violated in our models.

Statistical Colocalisation

We investigated whether the *HFE* p.C282Y rare homozygous associations with each prevalent disease could be due to statistical colocalisation with other genetic variants in the region. There were 33,171 variants available in the UK Biobank imputed genotype data (v3) within 500kb of p.C282Y (rs1800562, chr 6, hg19/b37 position 26093141). Genotypes with imprecise imputation were first recoded using the same criteria as for p.C282Y (see Supplementary Methods). We analysed those that had imputation quality >0.4, Hardy-Weinberg equilibrium p-value $>1*10^{-12}$, and minor allele frequency >1% (irrespective of correlation with p.C282Y). Variants with fewer than 3 homozygous-rare cases in the analysis sample could not be analysed; therefore analyses of more common conditions included more variants. Using logistic regression models, as described previously, we determined the association between homozygous rare and homozygous common participants with baseline p.C282Y-associated conditions: in males, haemochromatosis (*n*=1,854 genetic variants included), coronary heart disease (*n*=3,572 variants), diabetes (type 1 or 2) (*n*=3,302 variants), osteoarthritis (*n*=3,575 variants), osteoporosis (*n*=2,986 variants), pneumonia (*n*=3,189 variants), Rheumatoid arthritis (*n*=3,058 variants), and the combined phenotype of ≥1 diagnoses of all the aforementioned diseases (*n*=3,650 variants). In females, we analysed haemochromatosis (*n*=1,041 genetic variants included), osteoarthritis (*n*=3,620 variants), and the combined phenotype of ≥1 diagnoses of all associated diseases (*n*=3,596 variants). These analyses were repeated including p.C282Y as a covariate, to identify whether genetic variants in the locus had independent effects. LocusZoom (http://www.locuszoom.org) was used to plot the region [4], with Linkage Disequilibrium from “hg19/1000 Genomes Nov 2014 EUR” data.

Supplementary Results

Proportional hazards assumption

The global test for violation was not significant ($p<0.05$) for the models assessing p.C282Y status and time to our 11 main outcomes, with only one exception: for diabetes diagnosis in males, the assumption was violated (p=0.0091). Supplementary Figure 6 shows the Kaplan-Meier survival curve for this model, and the deviation in proportional hazards can be observed in the period from baseline to approximately 2 years post assessment, during which few incident cases of diabetes were diagnosed. After excluding the first 2 years of data (n=3,447 of 205,816 participants) the association between p.C282Y homozygosity and incident diabetes in males was still significant (HR=1.70, 95% CI 1.30 to 2.21, p=0.0001) and there was no longer significant violation of the proportional hazards assumption (p=0.13) – see Supplementary Figure 7 for Kaplan Meier plot.

Comparing Cox's proportional hazards to competing risks regression

In sensitivity analyses (Supplementary Table 9) we performed competing risk regression models including mortality as a competing risk, as previous evidence suggested that many cases of heamochromatosis are only diagnosed late in life, when background mortality is substantial.

In male p.C282Y homozygotes, the sub-Hazard Ratio from the competing risks regression model for Hazards of incident haemochromatosis was 284.6 (95% CIs 210.2 to 385.4), compared to a Hazard Ratio of 286 (95% CIs 211 to 388) from a Cox's proportional hazard model, for the same analysis. In competing risks regression models the male p.C282Y homozygotes the sub-Hazard Ratio for ≥1 incident diagnosis (of haemochromatosis itself, any liver disease (including liver cancer), diabetes, osteoarthritis or rheumatoid arthritis) was sHR=3.35 (95% CIs 2.84 to 3.95), which compares to a Hazard Ratio of HR=3.37 (95% CI 2.87 to 3.96) from a Cox's proportional hazards regression model.

Statistical Colocalisation

The associations between the p.C282Y missense mutation with each disease were as described in the 'Prevalent Conditions' results section of the paper. Co-inherited genetic variants ($R^2>0.8$) in the locus were also associated with these diagnoses, with seven variants at modestly greater statistical significance (smaller p-value) than p.C282Y itself (see Supplementary Figures 15-26, and Supplementary Table 12). However, after adjustment for p.C282Y genotype, no co-inherited variants remained significant ($p>0.05$: see Supplementary Figures 15-26, and Supplementary Table 12). All 7 potential variants were intergenic or intronic (non-coding) and only two were directly genotyped on the microarrays (rs1408272 and rs80215559): these two were reported in GWAS to be associated with transferrin saturation[5] and iron binding capacity[6], respectively, but no associations with specific diagnoses were reported (EBI GWAS catalogue[7] searched 29th October 2018): it may be that these two variants are tagging the known effect of p.C282Y on iron absorption.

Other genetic variants in the locus were also associated with the diseases, however as all of these were not co-inherited with p.C282Y ($R^2<0.2$), they cannot explain the statistical associations between p.C282Y and the diseases, despite being at the same locus.

Overall, we therefore found no strong evidence for the various *HFE* p.C282Y disease associations being caused by other variants in the locus. However, most of the potential candidates were not directly genotyped, so further work may be justified with directly genotyped (sequencing) data.

Supplementary Figures

Supplementary Figure 1 – Prevalent conditions by age-subgroup and sex: Forest plot of associations (Odds Ratios) comparing p.C282Y homozygous status to wild type (no p.C282Y) in A) males and B) females separately.

Logistic regressions adjusted for age, genotyping array and ancestry principal components 1-5. CIs=95% Confidence Intervals. Full results including Coronary Artery Disease and atrial fibrillation results are available in Supplementary Table 1. * = too few cases for analysis.

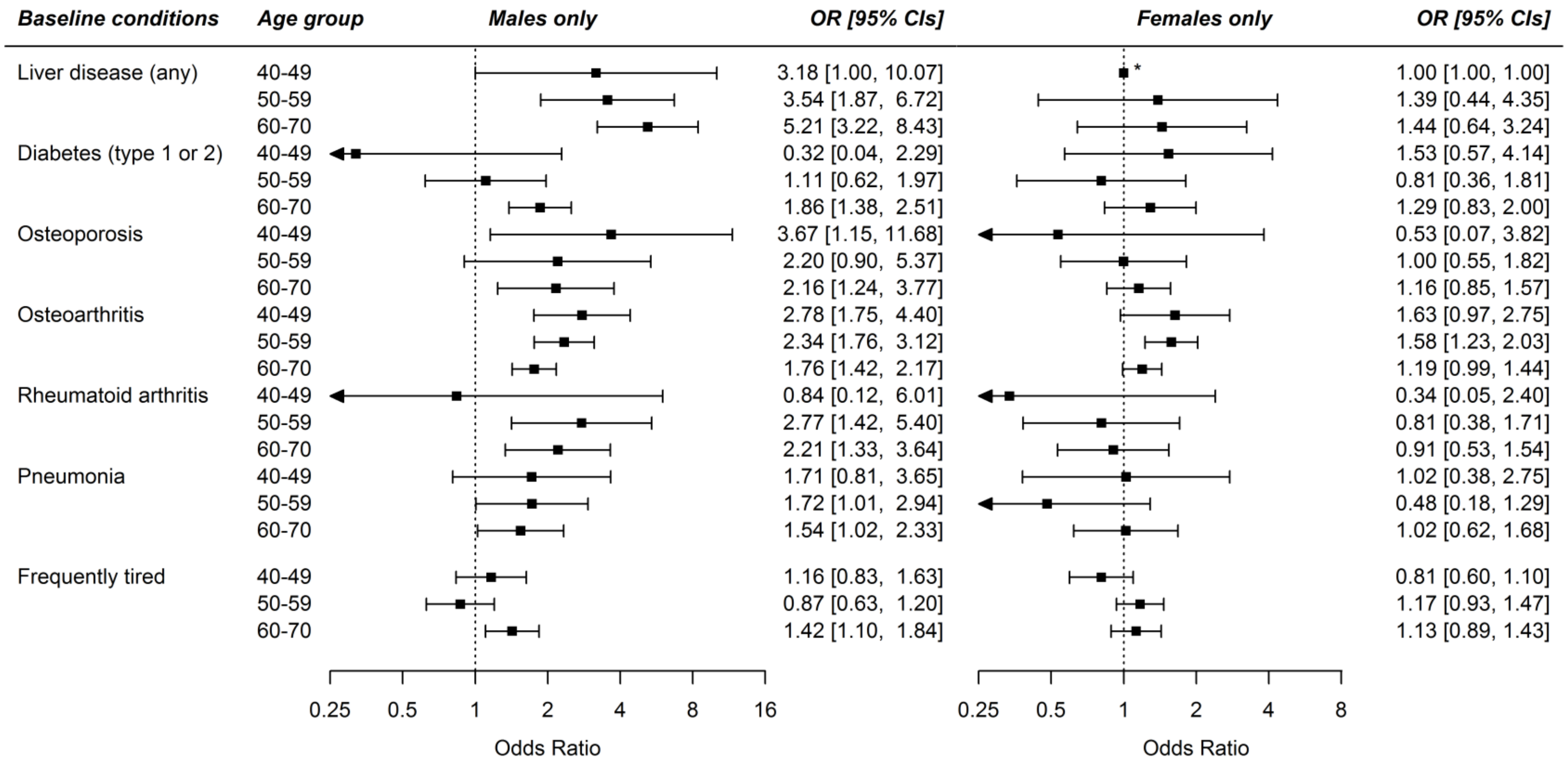

Supplementary Figure 2 – survival curve for p.C282Y genotypes and mortality

p.C282Y homozygous genotype was associated with mortality compared to those with no p.C282Y mutations, for both male and female participants aged 40-70 at baseline. This analysis was irrespective of p.H63D status.

Hazard Ratio 1.225, 95% CIs 1.012 to 1.482, p=0.037

See Methods and Supplementary Table 3 for further details.

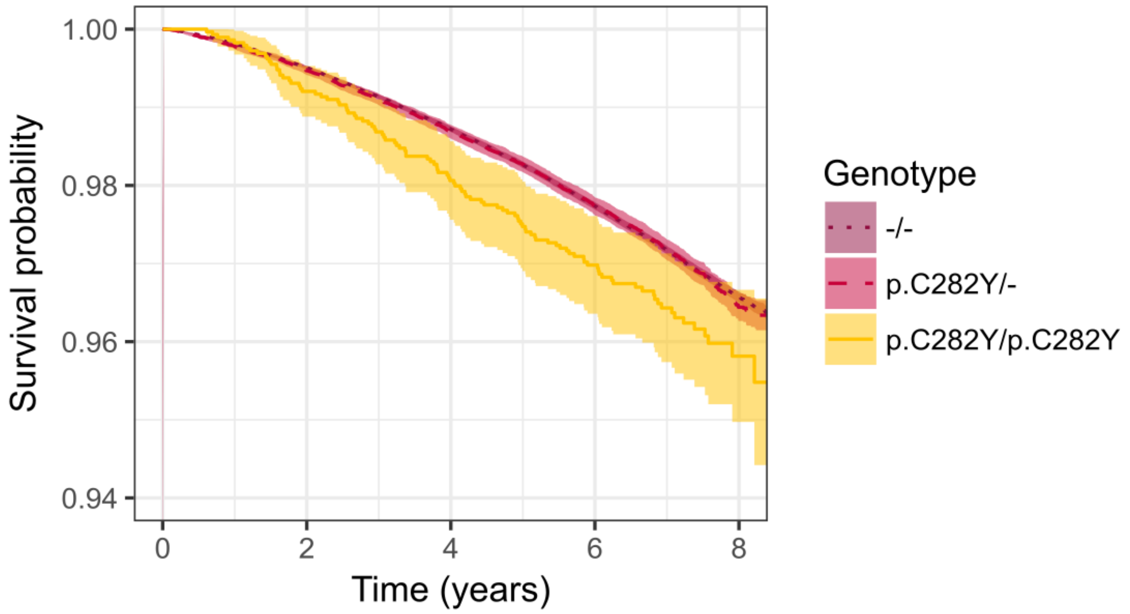

Supplementary Figure 3 – survival curve for p.C282Y genotype and any incident diagnosis (males)

p.C282Y homozygous genotype was associated with increased likelihood of incident diagnosis of at least one baseline-associated morbidity (haemochromatosis, liver disease, liver cancer, osteoarthritis, rheumatoid arthritis, or diabetes (type 1 or type 2), in males aged 40-70, excluding participants with a diagnosis at baseline, compared to those with no p.C282Y mutations (irrespective of p.H63D status, n=0 for p.C282Y homozygosity and p.H63D mutations). Hazard Ratio 3.37 95% CIs 2.87 to 3.97, p=$1*10^{-48}$. See Methods and Supplementary Table 4 for details

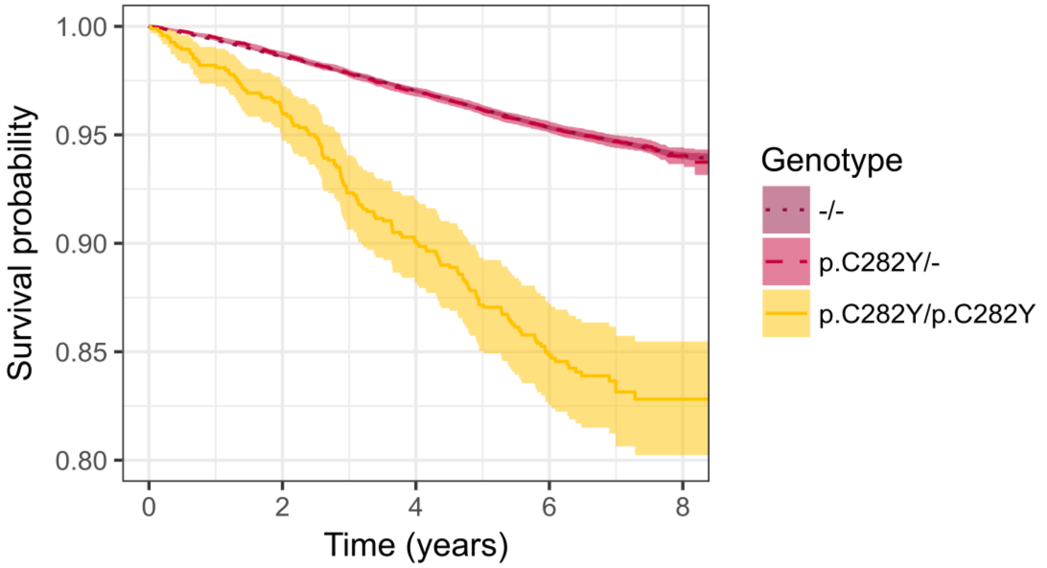

Supplementary Figure 4 – survival curve for p.C282Y genotype and any incident diagnosis (females)

As above, in female participants only. Hazard Ratio 2.99 95% CIs 2.51 to 3.55, p=$7*10^{-35}$

See Methods and Supplementary Table 4 for details

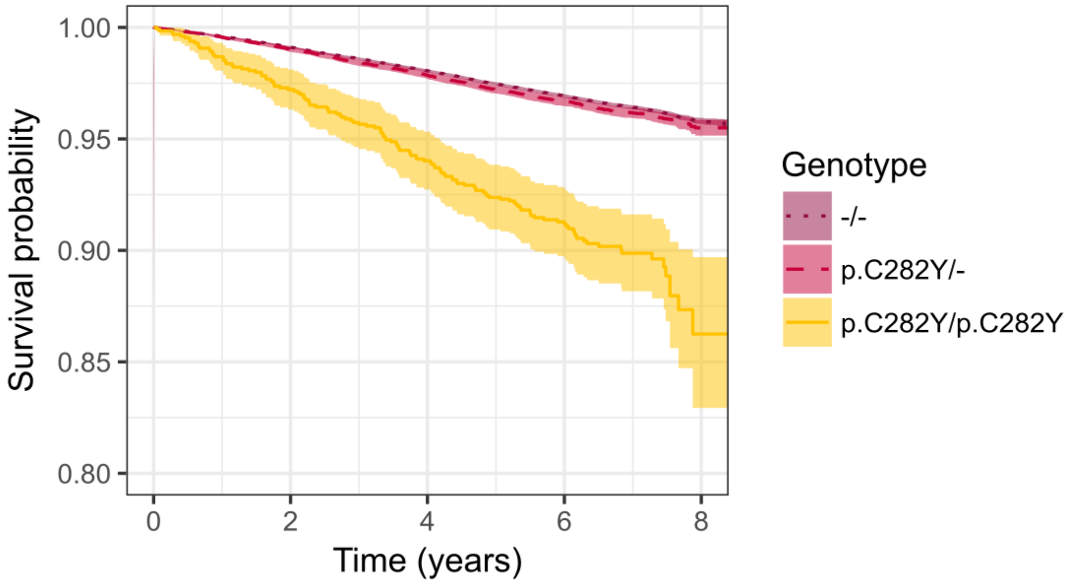

Supplementary Figure 5 – overall excess incident morbidity

p.C282Y genotype is associated with overall excess morbidity (at least one of haemochromatosis, liver disease, liver cancer, osteoarthritis, rheumatoid arthritis, or diabetes (type 1 or type 2) - prevalent and incident diagnoses combined) in male and female participants aged 40-70 (irrespective of p.H63D genotype).

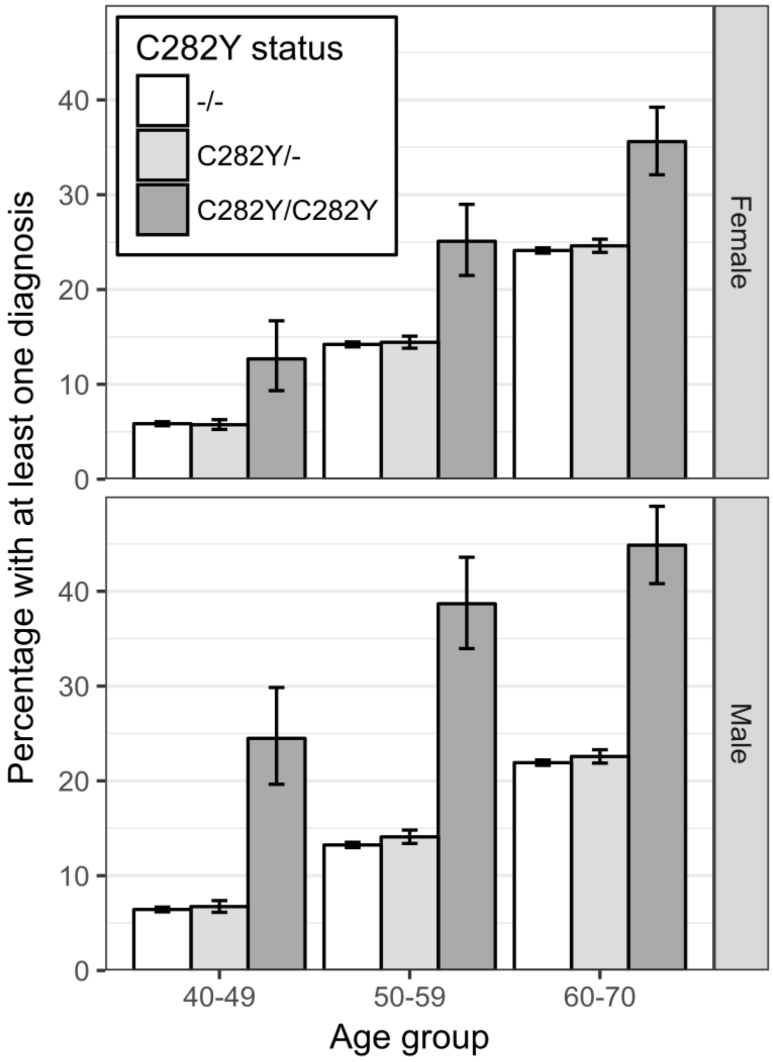

Supplementary Figure 6 – survival curve for p.C282Y genotype and incident diabetes (males)

p.C282Y homozygous genotype is associated with increased likelihood of incident diabetes (type 1 or type 2) in males aged 40-70. Hazard Ratio 1.45, 95% CIs 1.14 to 1.85, p=0.003. See Methods and Supplementary Table 4 for details. We identified a statistically significant violation in the proportional hazards assumption for this model (p=0.0091). See Supplementary Information and Supplementary Figure 7 for more details.

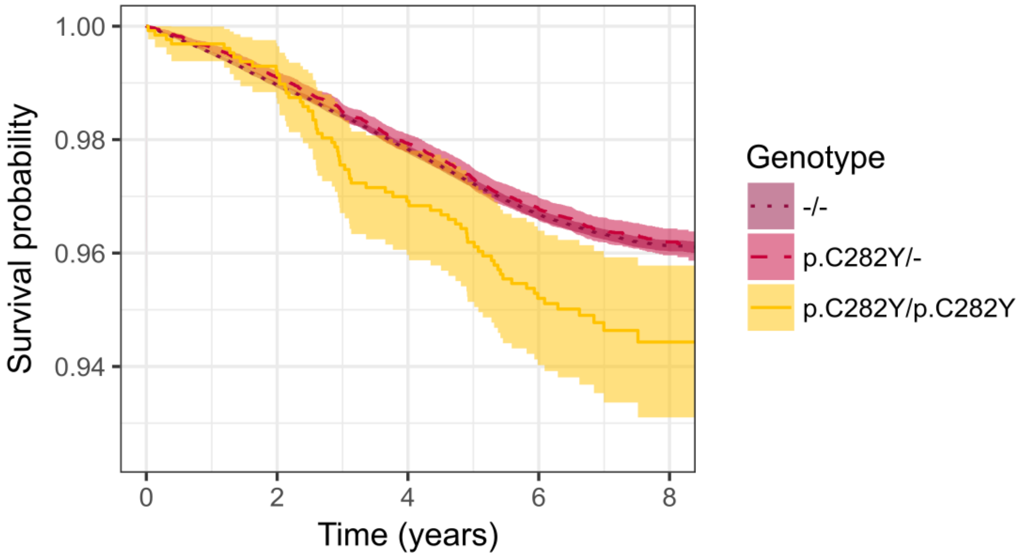

Supplementary Figure 7 – survival curve for p.C282Y genotype and incident diabetes (males) excluding first 2 years

p.C282Y homozygous genotype is associated with increased likelihood of incident diabetes (type 1 or type 2) in males aged 40-70. There is no longer a significant violation of the proportional hazards assumption when excluding the first 2 years of follow up (p>0.05). Hazard Ratio 1.70, 95% CI 1.30 to 2.21, p=0.0001

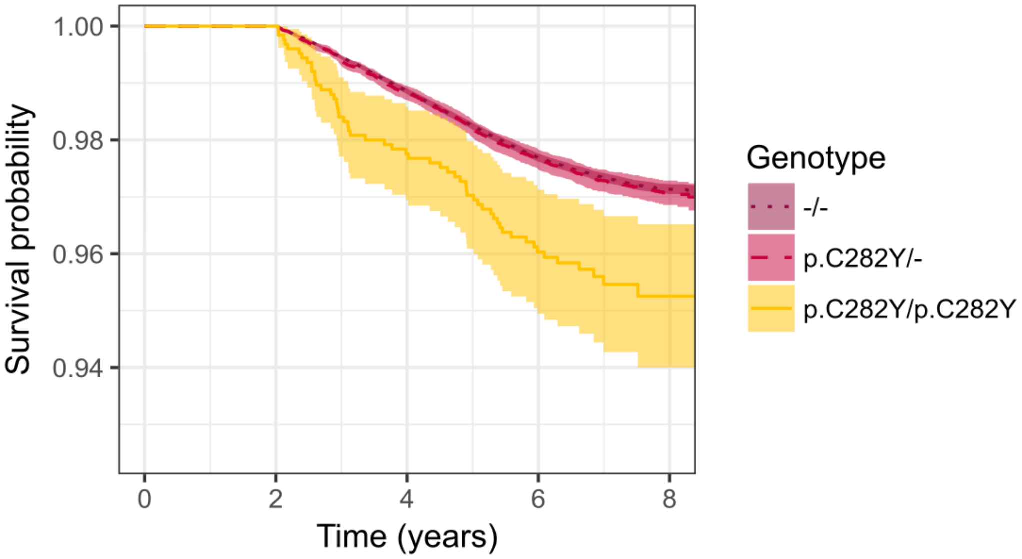

Supplementary Figure 8 – MR analysis of transferrin saturation and haemochromatosis in males

Plot of HFE genotype effect on Transferrin Saturation (x-axis) and risk of incident haemochromatosis (y-axis) in males. Mendelian Randomization (MR) regression results are shown, which provide an estimate of the causal effect. Data input and full results in Supplementary Tables 10 and 11 (IVW = inverse variance weighted).

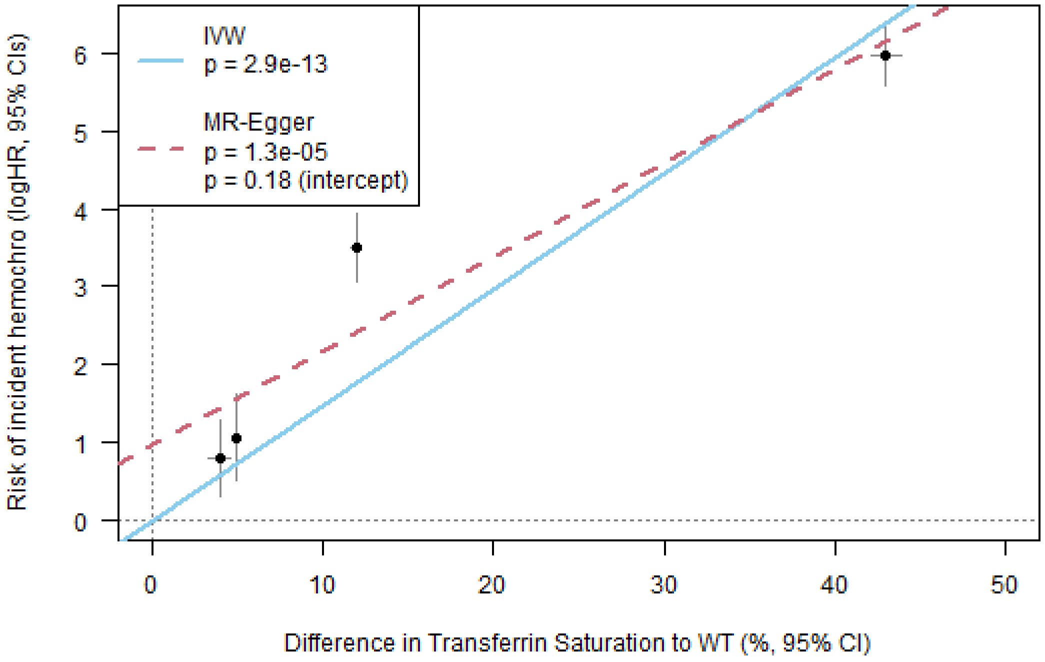

Supplementary Figure 9 – MR analysis of transferrin saturation and liver disease in males

Plot of HFE genotype effect on Transferrin Saturation (x-axis) and risk of incident liver disease (y-axis) in males. Mendelian Randomization (MR) regression results are shown, which provide an estimate of the causal effect. Data input and full results in Supplementary Tables 10 and 11 (IVW = inverse variance weighted).

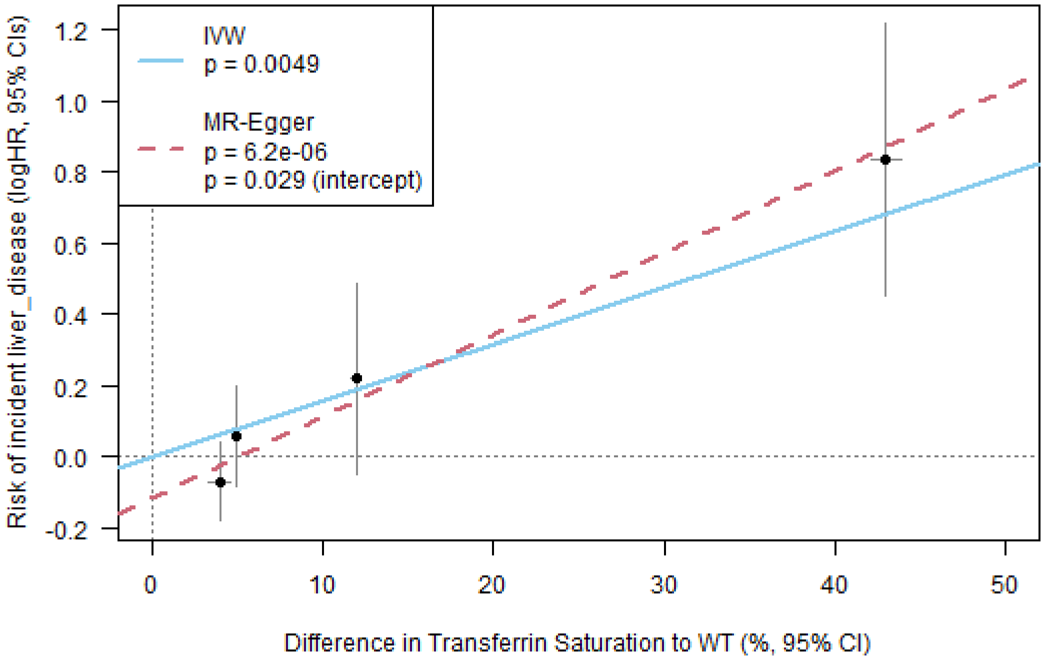

Supplementary Figure 10 – MR analysis of transferrin saturation and liver cancer in males

Plot of HFE genotype effect on Transferrin Saturation (x-axis) and risk of incident liver cancer (y-axis) in males. Mendelian Randomization (MR) regression results are shown, which provide an estimate of the causal effect. Data input and full results in Supplementary Tables 10 and 11 (IVW = inverse variance weighted).

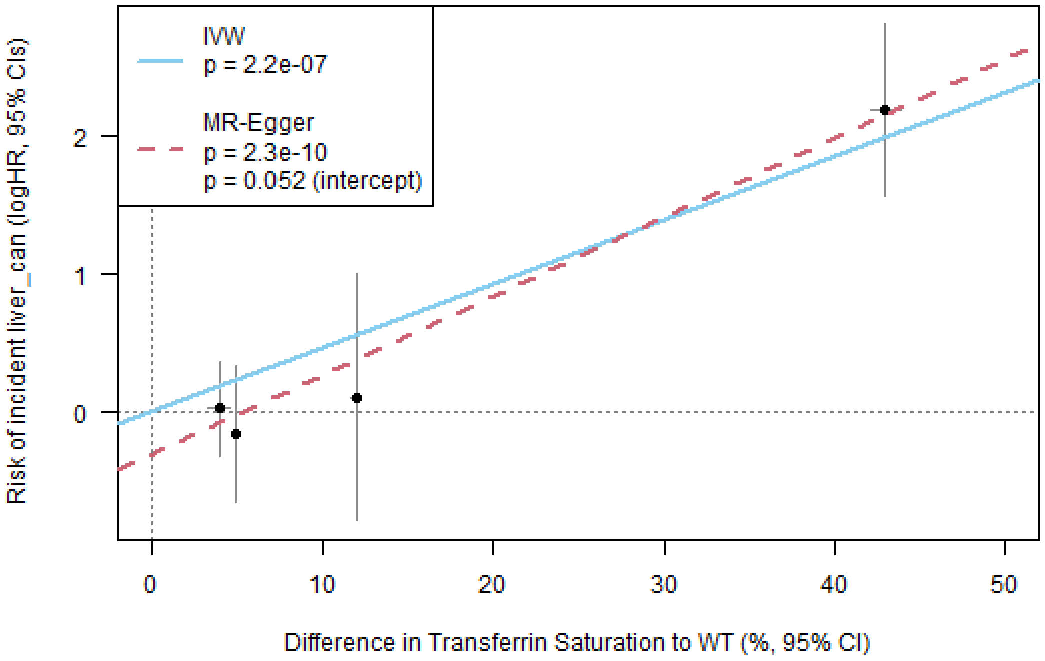

Supplementary Figure 11 – MR analysis of transferrin saturation and osteoarthritis in males

Plot of HFE genotype effect on Transferrin Saturation (x-axis) and risk of incident osteoarthritis (y-axis) in males. Mendelian Randomization (MR) regression results are shown, which provide an estimate of the causal effect. Data input and full results in Supplementary Tables 10 and 11 (IVW = inverse variance weighted).

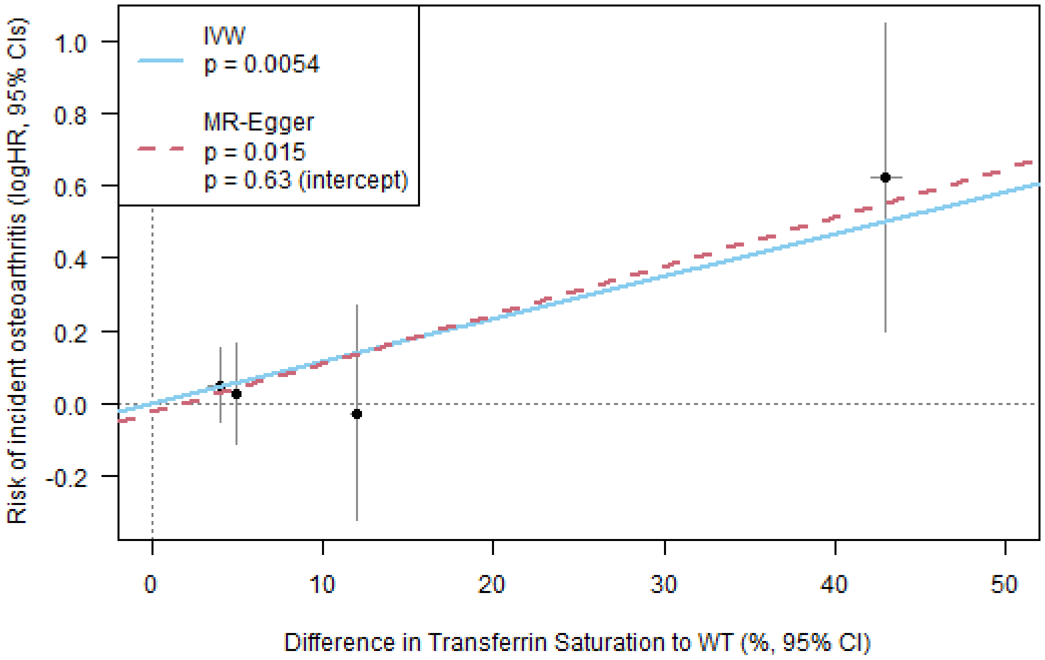

Supplementary Figure 12 – MR analysis of transferrin saturation and osteoporosis in males

Plot of HFE genotype effect on Transferrin Saturation (x-axis) and risk of incident osteoporosis (y-axis) in males. Mendelian Randomization (MR) regression results are shown, which provide an estimate of the causal effect. Data input and full results in Supplementary Tables 10 and 11 (IVW = inverse variance weighted).

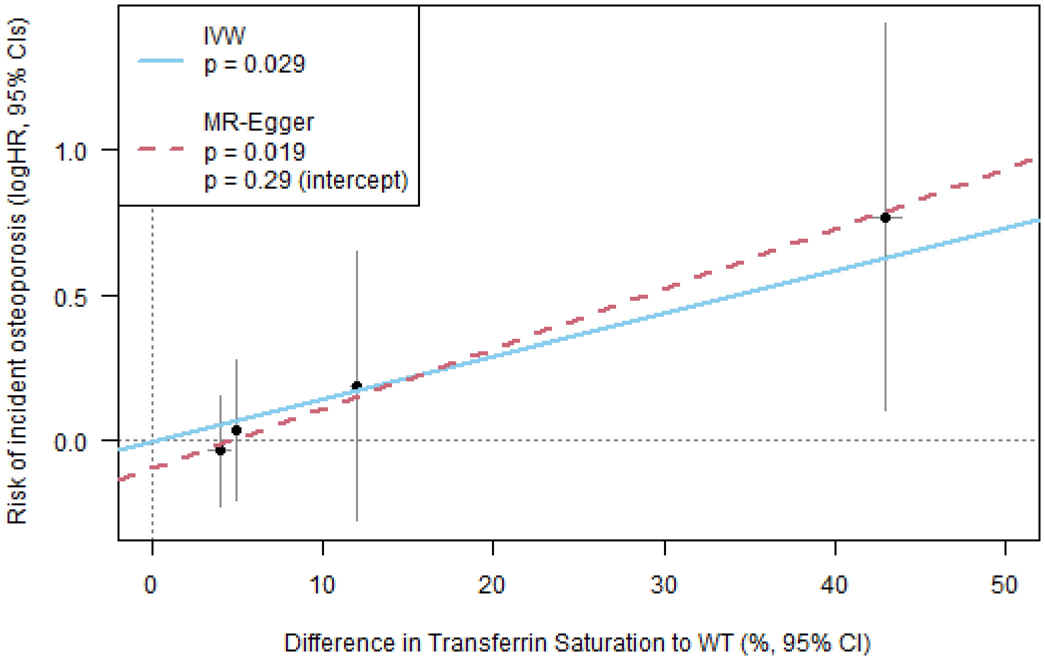

Supplementary Figure 13 – MR analysis of transferrin saturation and haemochromatosis in females

Plot of HFE genotype effect on Transferrin Saturation (x-axis) and risk of incident haemochromatosis (y-axis) in females. Mendelian Randomization (MR) regression results are shown, which provide an estimate of the causal effect. Data input and full results in Supplementary Tables 10 and 11 (IVW = inverse variance weighted).

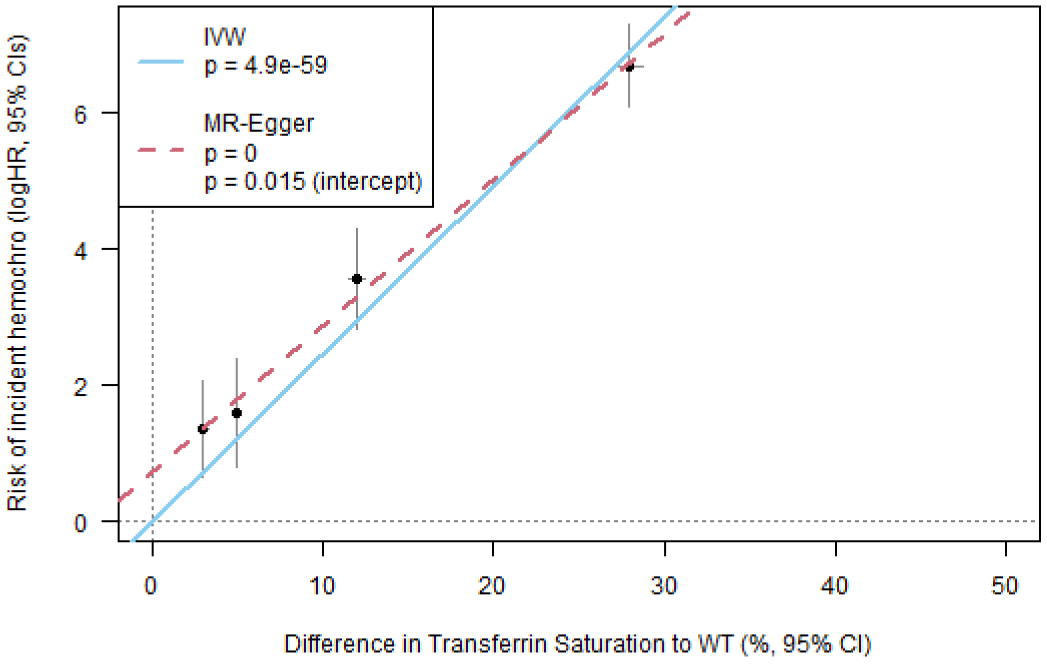

Supplementary Figure 14 – MR analysis of transferrin saturation and osteoarthritis in females

Plot of HFE genotype effect on Transferrin Saturation (x-axis) and risk of incident osteoarthritis (y-axis) in females. Mendelian Randomization (MR) regression results are shown, which provide an estimate of the causal effect. Data input and full results in Supplementary Tables 10 and 11 (IVW = inverse variance weighted).

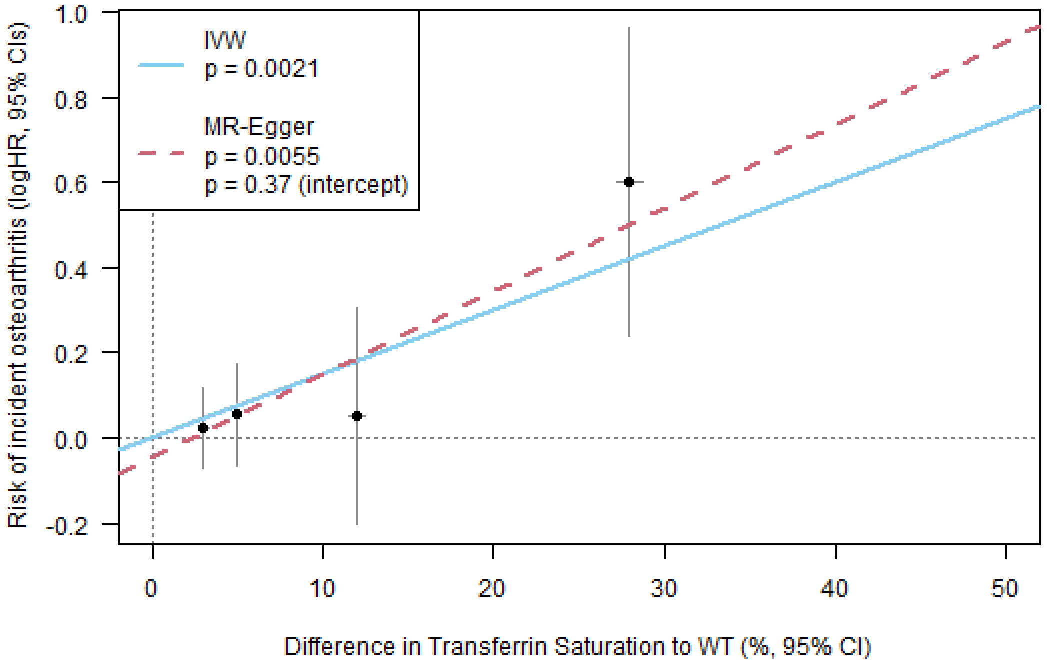

Supplementary Figure 15 – Colocalisation analysis of prevalent haemochromatosis in males

A) LocusZoom plot of genotypes +/- 500kb of p.C282Y and their association with prevalent haemochromatosis in males: y-axis = $-\log_{10}$(p-value). B) Shows the results with adjustment for p.C282Y genotype.

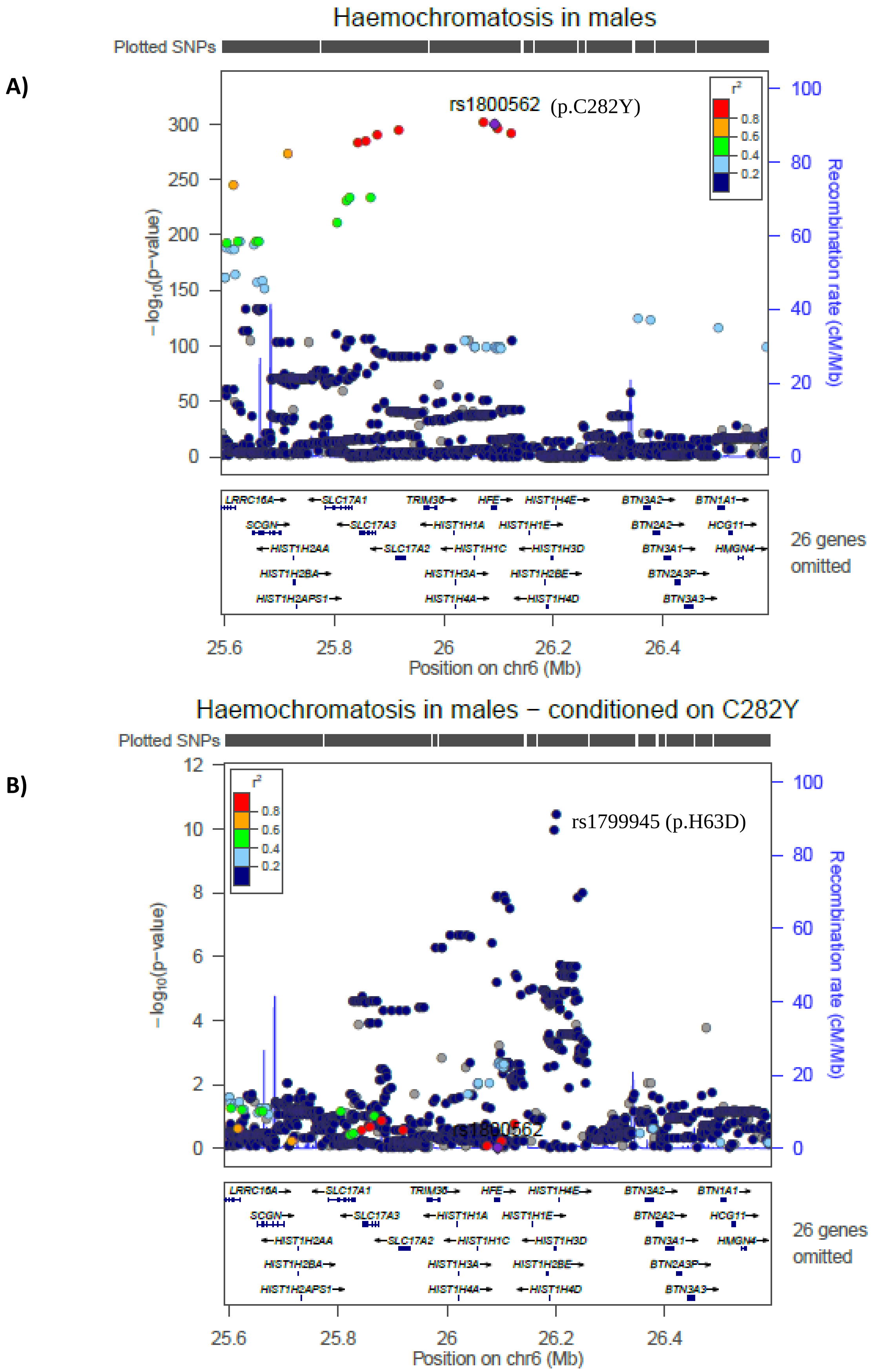

Supplementary Figure 16 – Colocalisation analysis of prevalent coronary heart disease (CHD) in males

A) LocusZoom plot of genotypes +/- 500kb of p.C282Y and their association with prevalent CHD in males: y-axis = $-\log_{10}$(p-value). B) Shows the results with adjustment for p.C282Y genotype.

A)

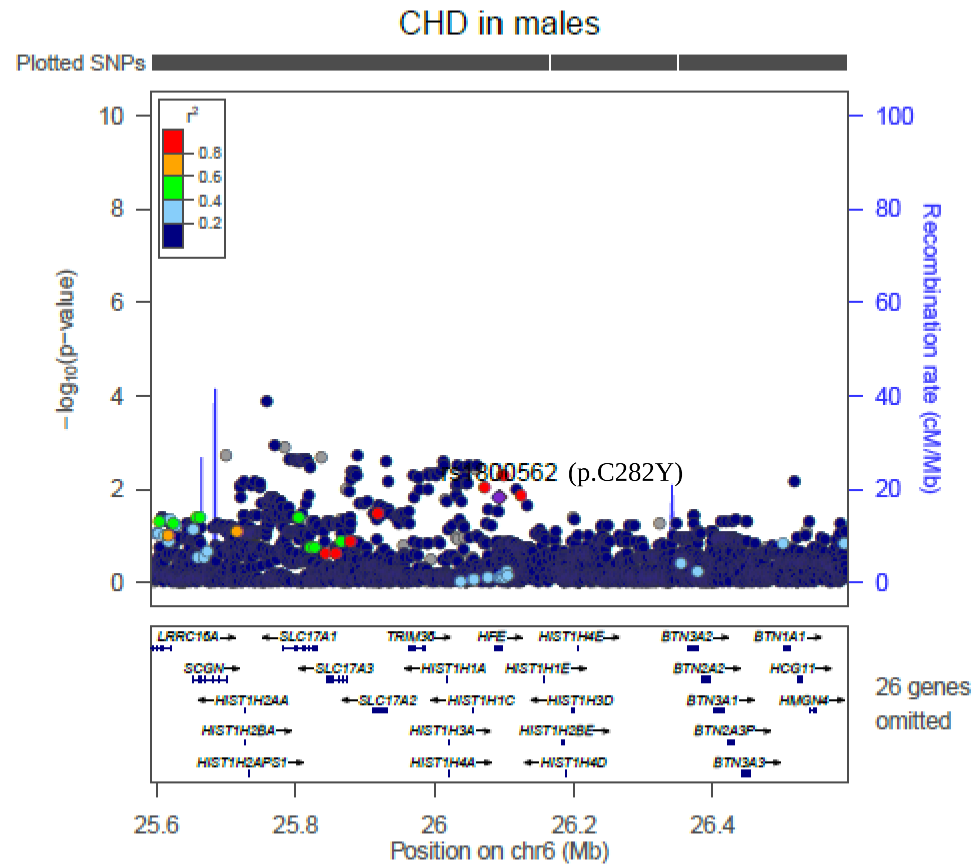

B)

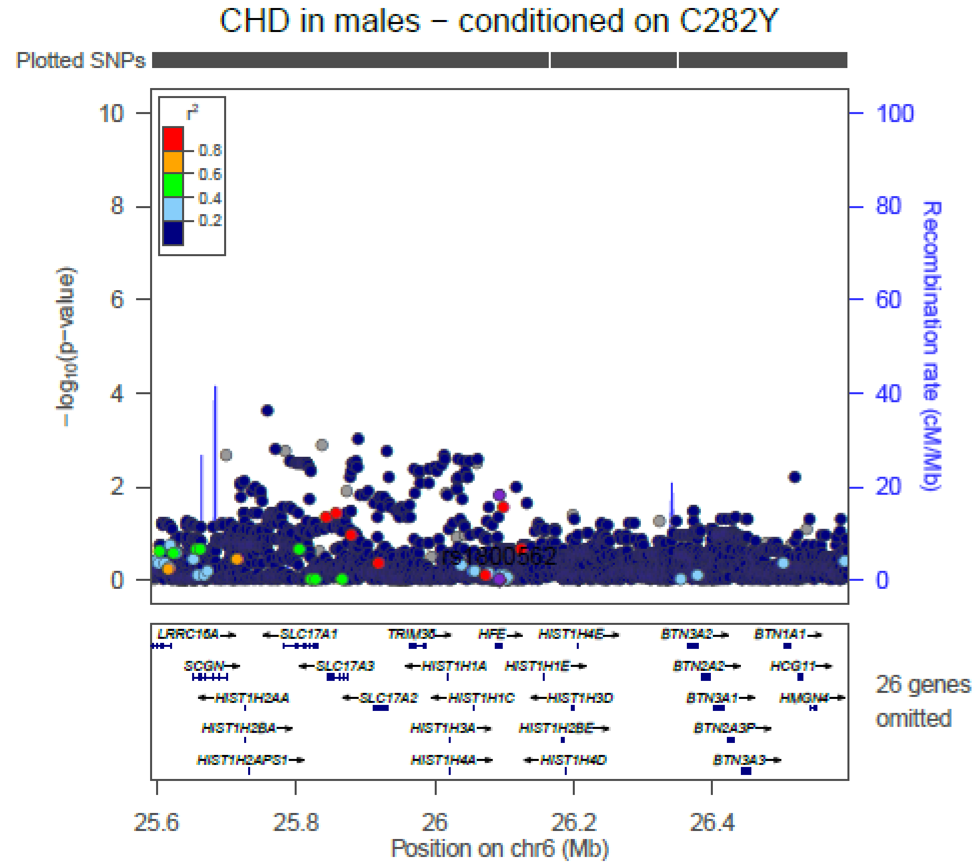

Supplementary Figure 17 – Colocalisation analysis of prevalent diabetes (type 1 or 2) in males

A) LocusZoom plot of genotypes +/- 500kb of p.C282Y and their association with prevalent diabetes (type 1 or 2) in males: y-axis = -$\log_{10}$(p-value). B) Shows the results with adjustment for p.C282Y genotype.

A)

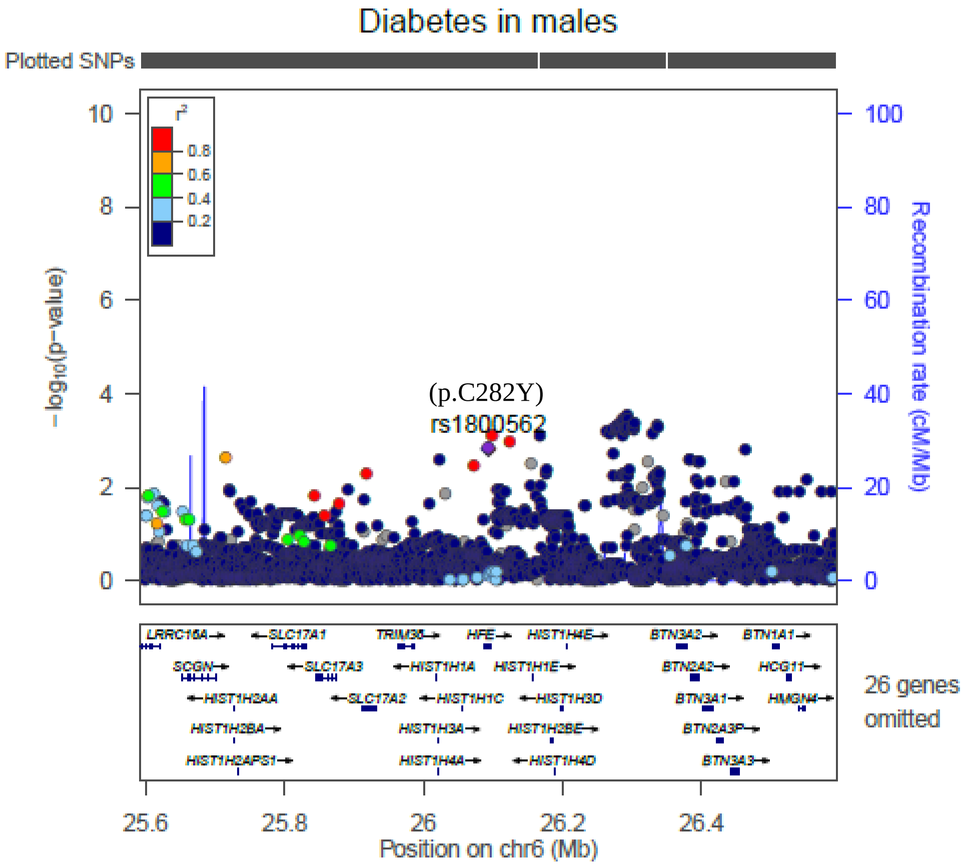

B)

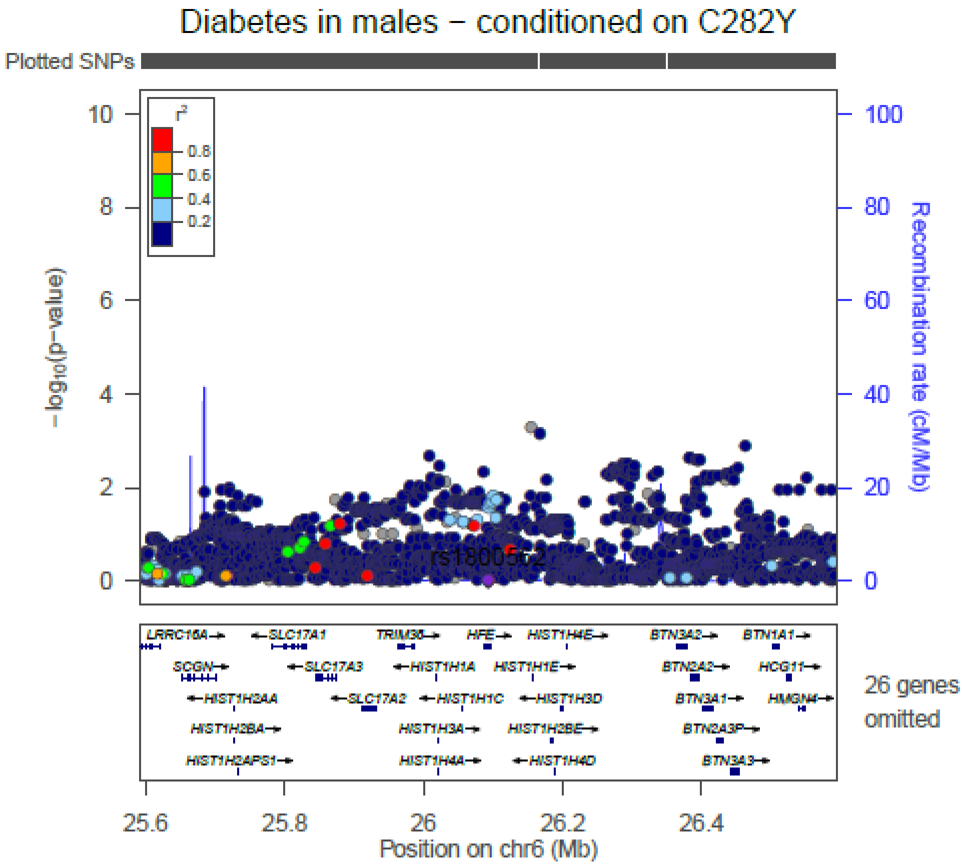

Supplementary Figure 18 – Colocalisation analysis of prevalent liver disease in males

A) LocusZoom plot of genotypes +/- 500kb of p.C282Y and their association with prevalent liver disease in males: y-axis = $-\log_{10}$(p-value). B) Shows the results with adjustment for p.C282Y genotype.

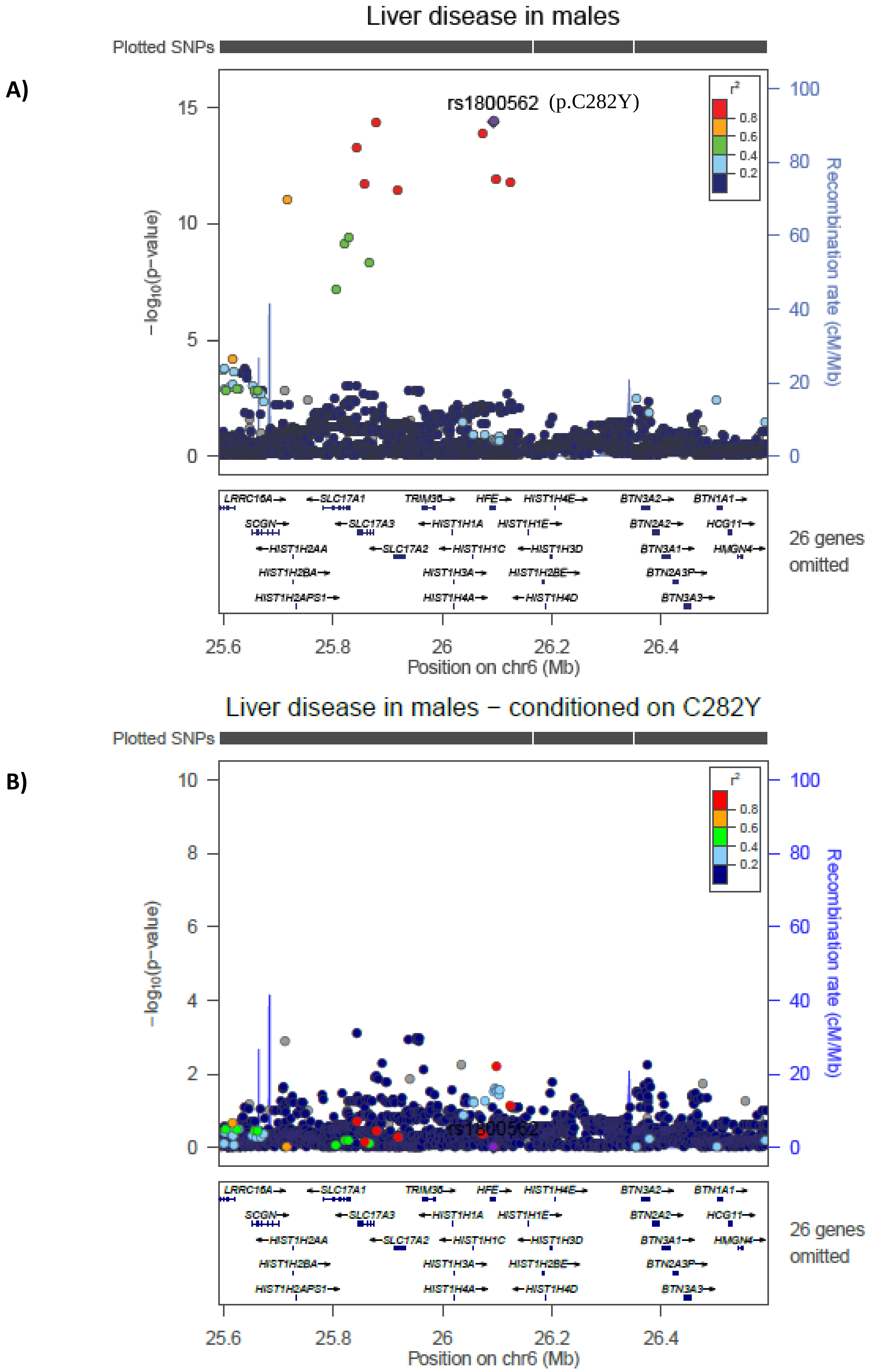

Supplementary Figure 19 – Colocalisation analysis of prevalent osteoarthritis in males

A) LocusZoom plot of genotypes +/- 500kb of p.C282Y and their association with prevalent osteoarthritis in males: y-axis = -log$_{10}$(p-value). B) Shows the results with adjustment for p.C282Y genotype.

A)

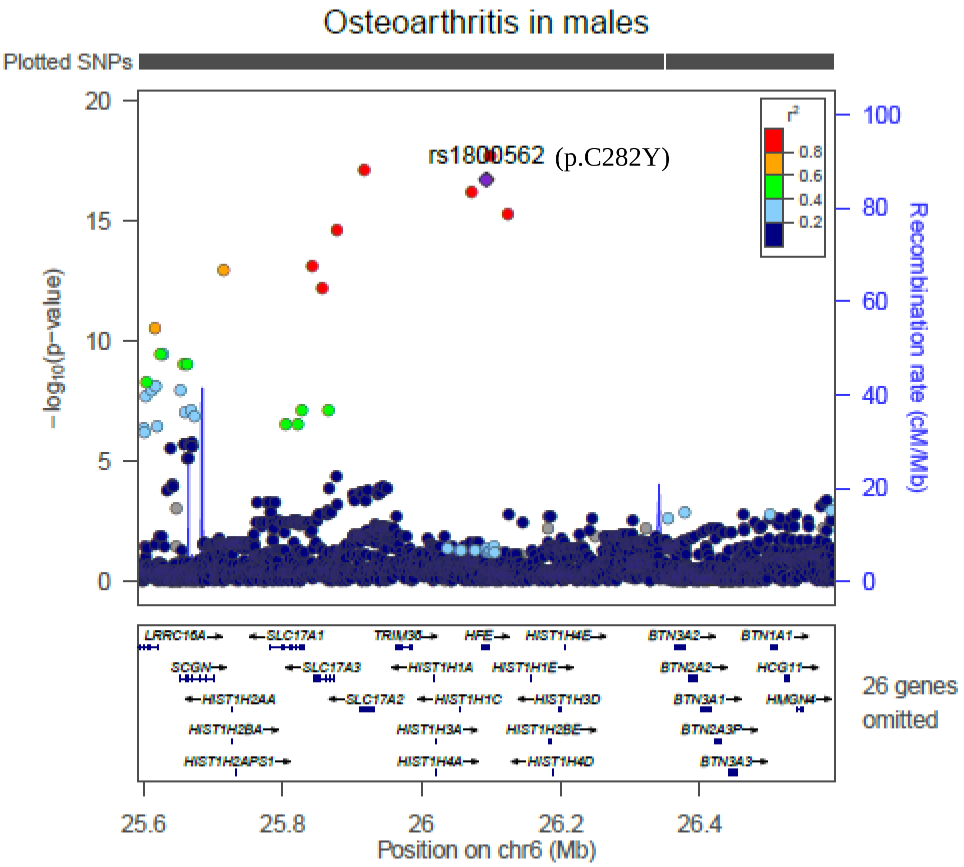

B)

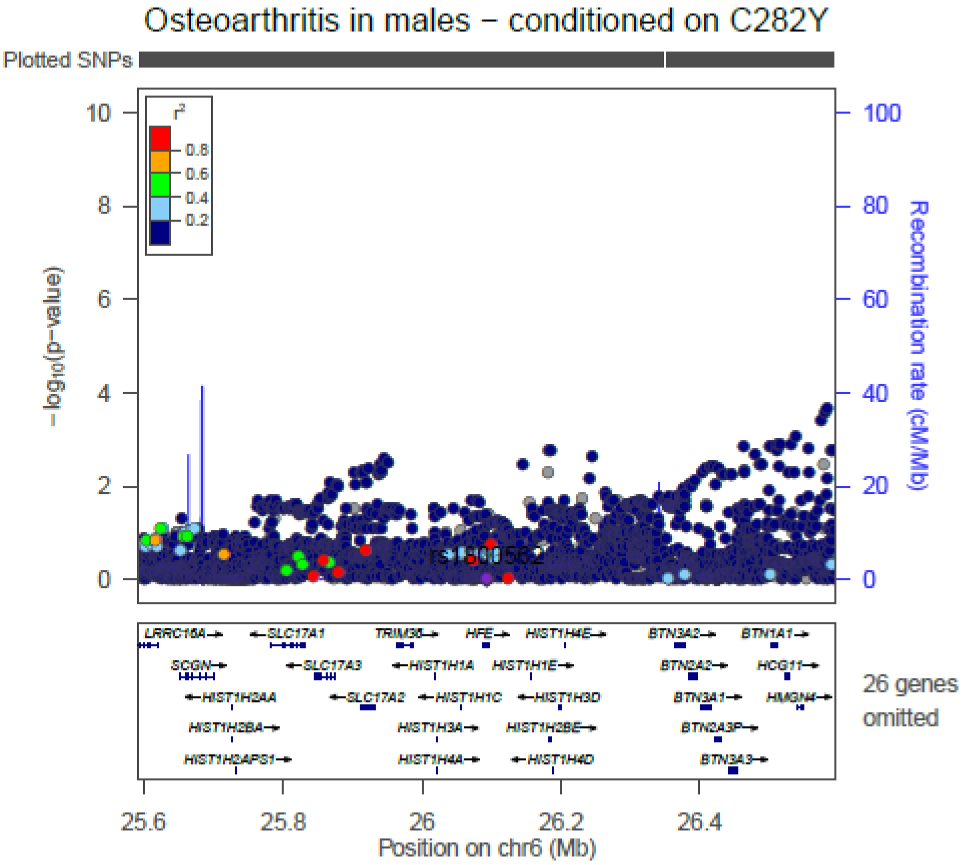

Supplementary Figure 20 – Colocalisation analysis of prevalent osteoporosis in males

A) LocusZoom plot of genotypes +/- 500kb of p.C282Y and their association with prevalent osteoporosis in males: y-axis = $-\log_{10}$(p-value). B) Shows the results with adjustment for p.C282Y genotype.

A)

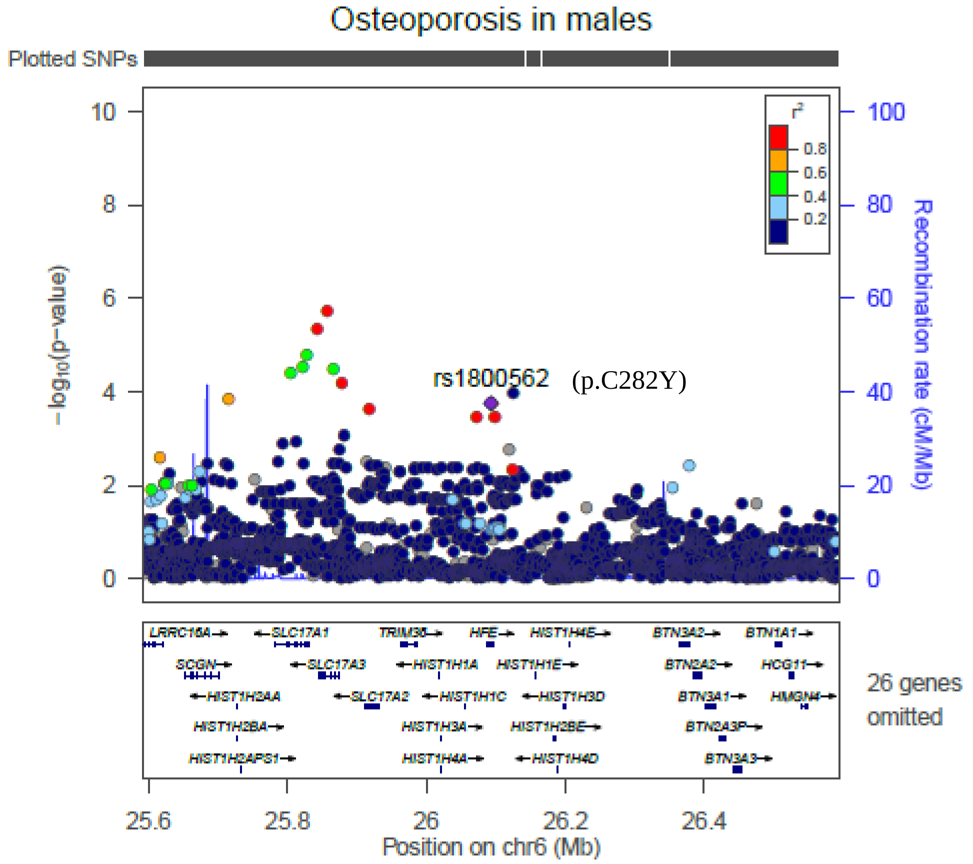

B)

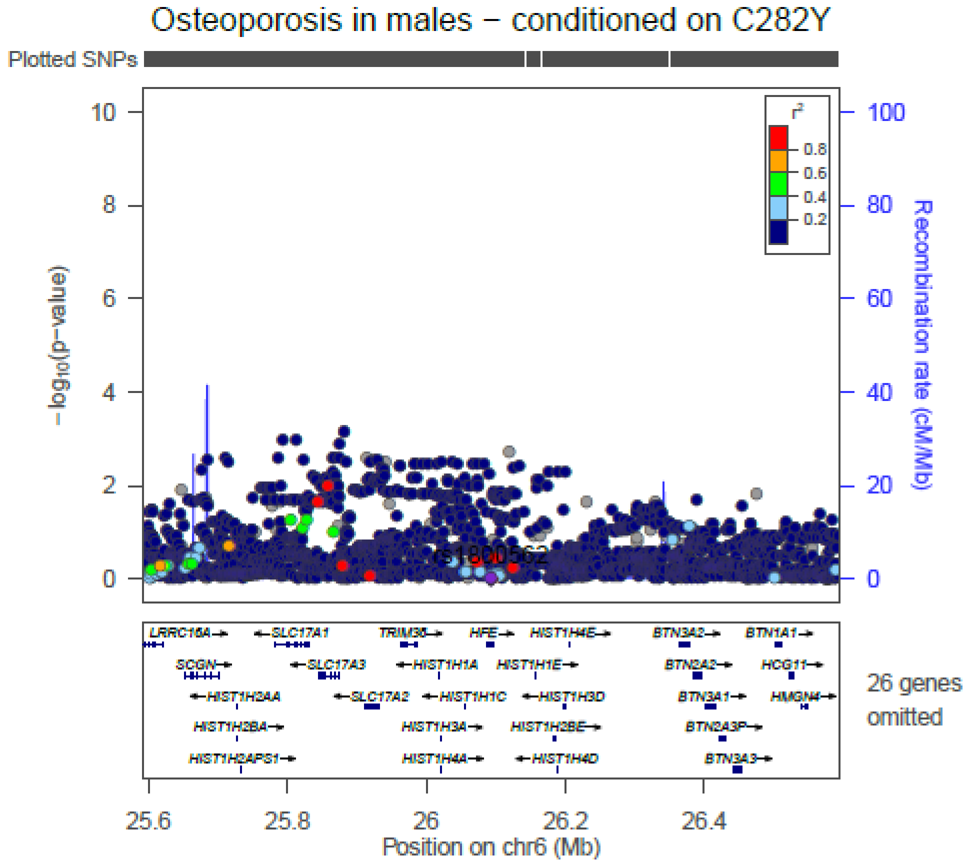

Supplementary Figure 21 – Colocalisation analysis of prevalent pneumonia in males

A) LocusZoom plot of genotypes +/- 500kb of p.C282Y and their association with prevalent pneumonia in males: y-axis = -$\log_{10}$(p-value). B) Shows the results with adjustment for p.C282Y genotype.

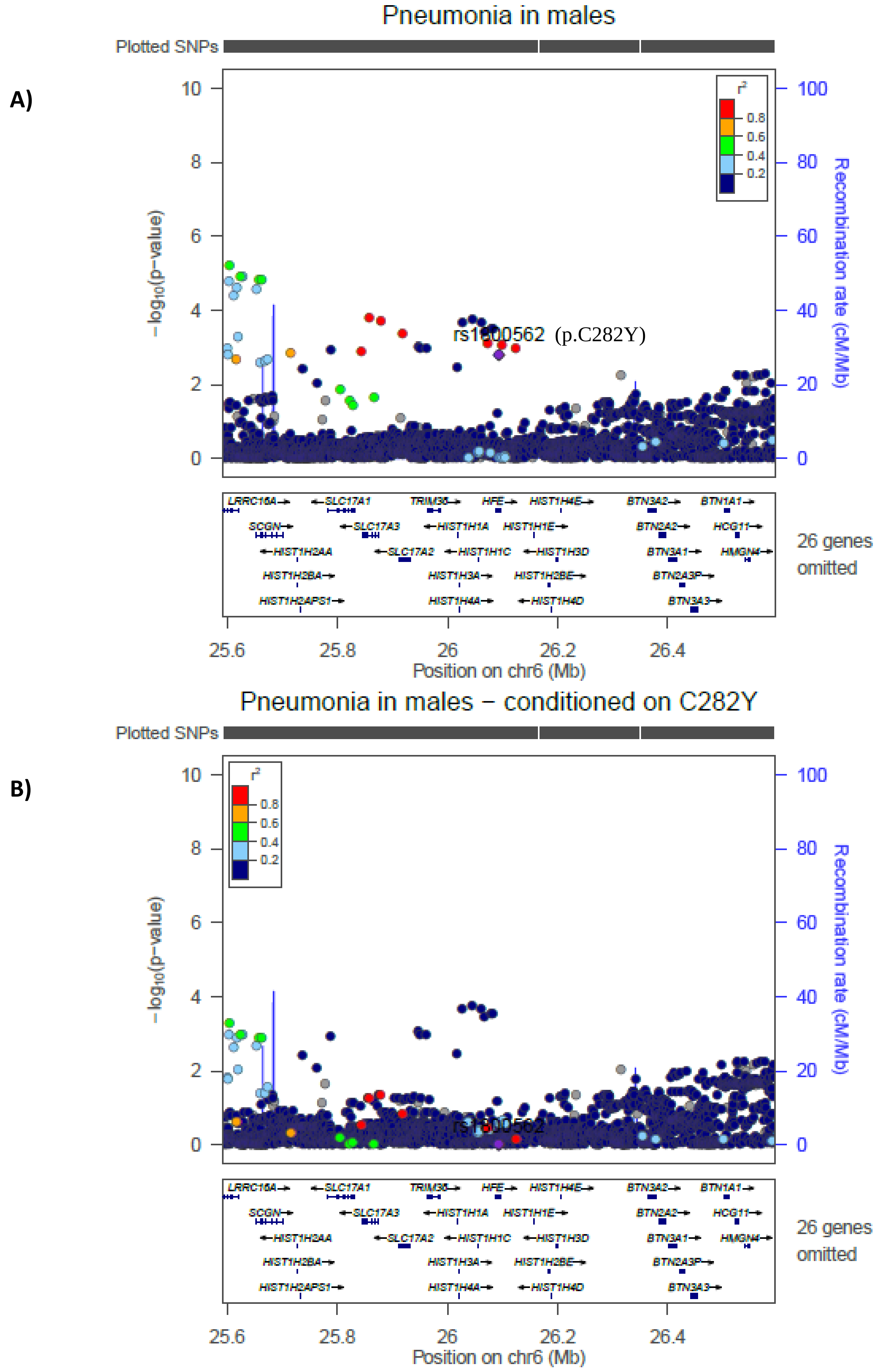

Supplementary Figure 22 – Colocalisation analysis of prevalent Rheumatoid arthritis in males

A) LocusZoom plot of genotypes +/- 500kb of p.C282Y and their association with prevalent Rheumatoid arthritis in males: y-axis = $-\log_{10}$(p-value). B) Shows the results with adjustment for p.C282Y genotype.

A)

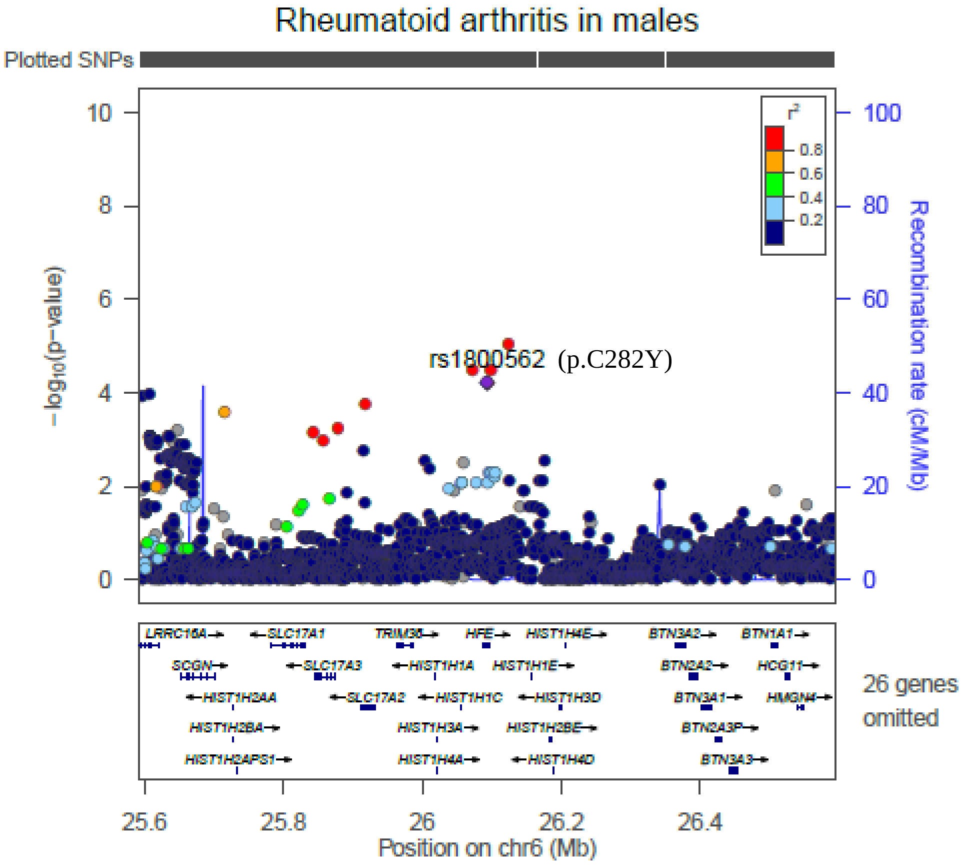

B)

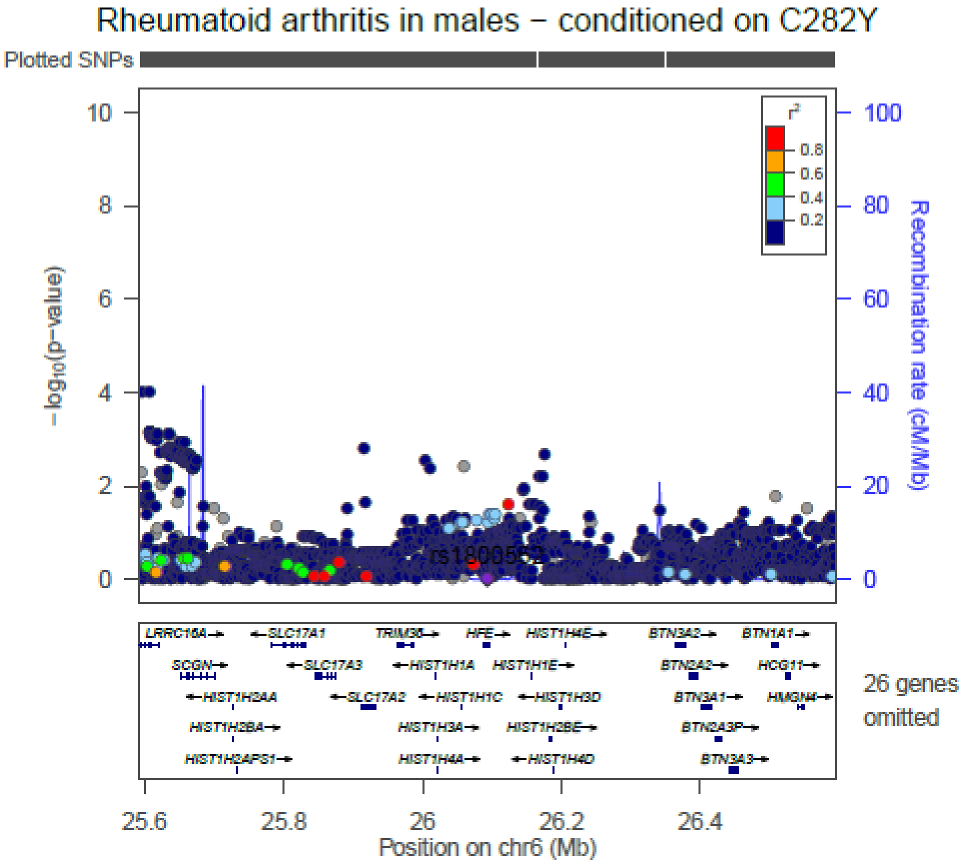

Supplementary Figure 23 – Colocalisation analysis of ≥1 associated prevalent diagnosis in males

A) LocusZoom plot of genotypes +/- 500kb of p.C282Y and their association with ≥1 diagnosis of haemochromatosis, CHD, diabetes, liver disease, osteoarthritis, osteoporosis, pneumonia, or Rheumatoid arhtirits in males: y-axis = -log$_{10}$(p-value). B) Shows the results with adjustment for p.C282Y genotype.

A)

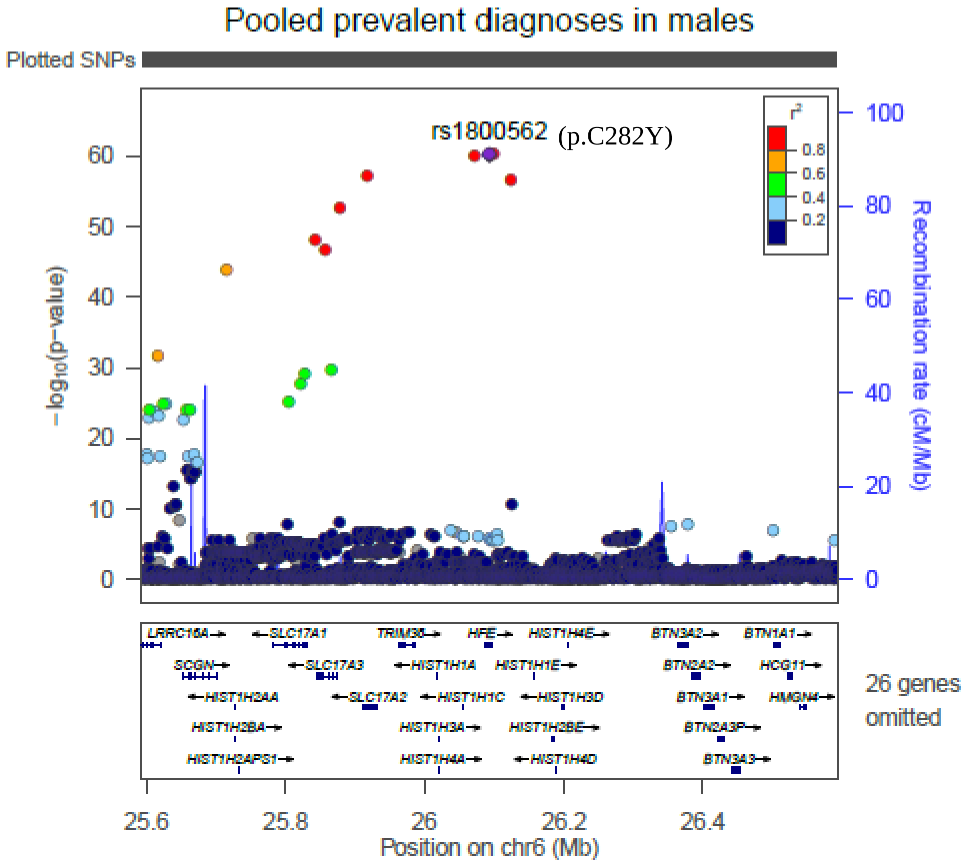

B)

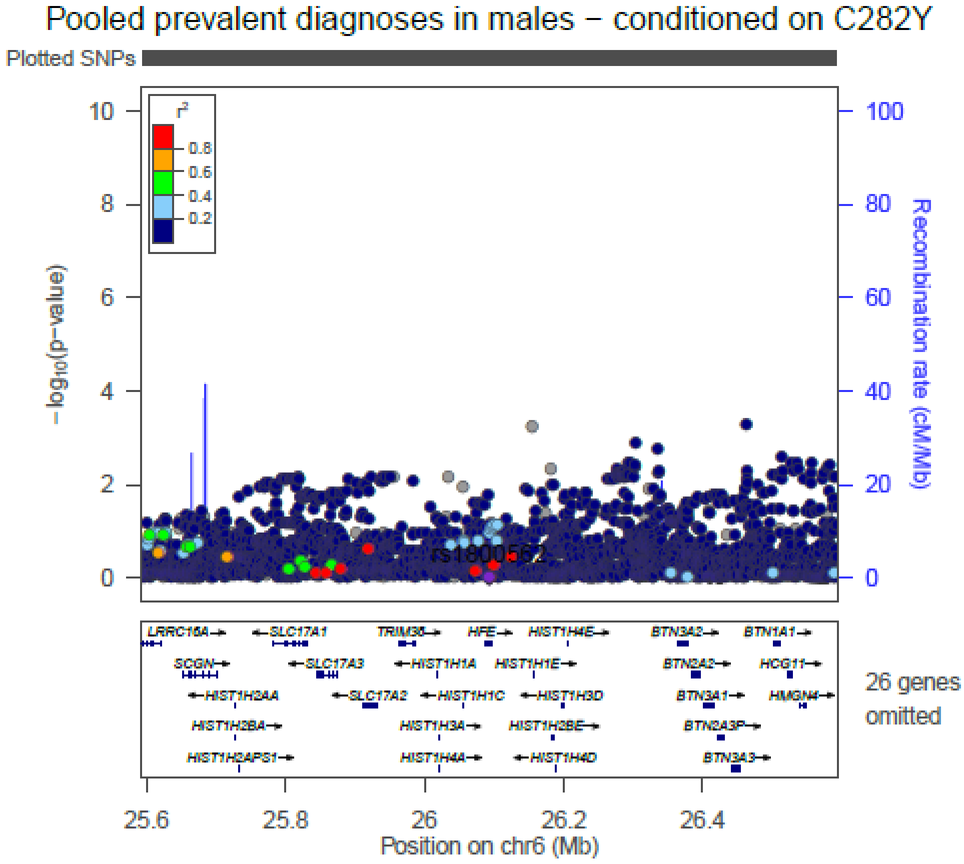

Supplementary Figure 24 – Colocalisation analysis of prevalent haemochromatosis in females

A) LocusZoom plot of genotypes +/- 500kb of p.C282Y and their association with haemochromatosis in females: y-axis = -$\log_{10}$(p-value). B) Shows the results with adjustment for p.C282Y genotype.

A)

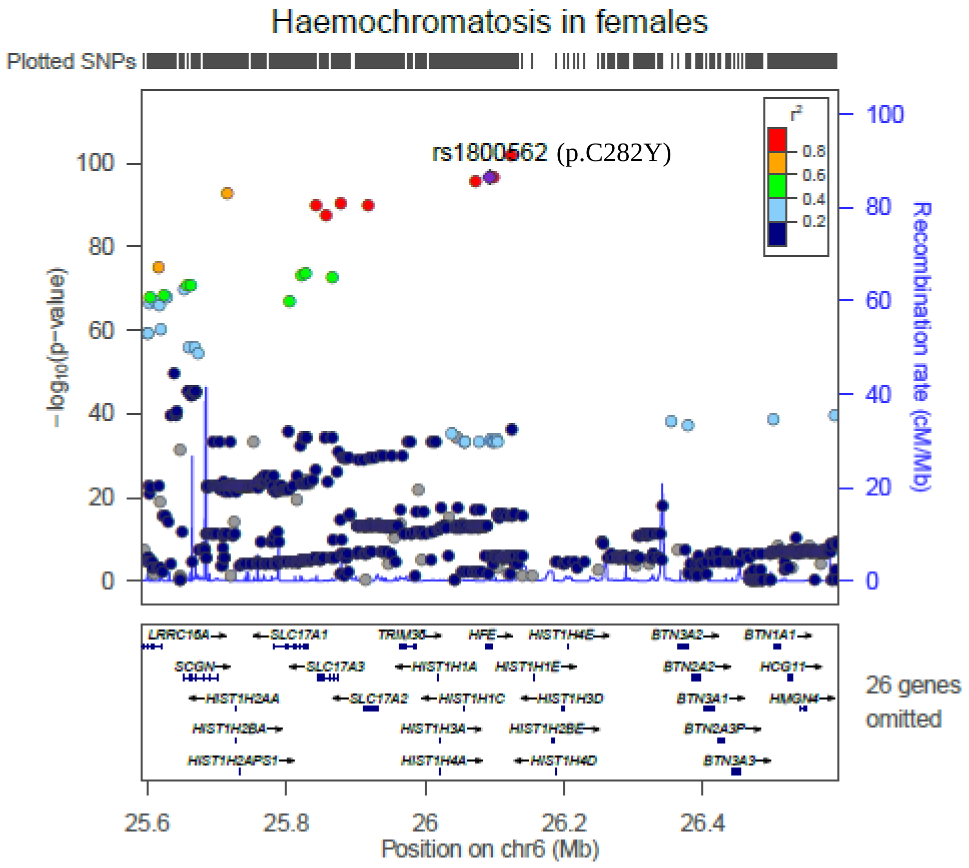

B)

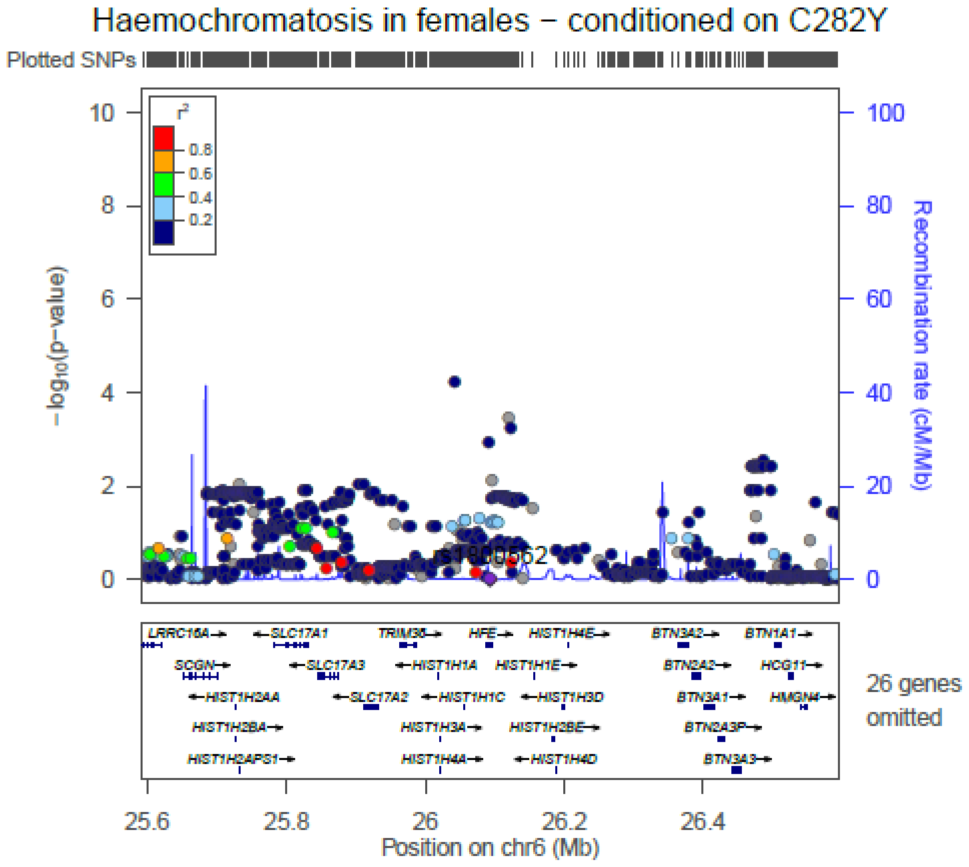

Supplementary Figure 25 – Colocalisation analysis of prevalent osteoarthritis in females

A) LocusZoom plot of genotypes +/- 500kb of p.C282Y and their association with osteoarthritis in females: y-axis = $-\log_{10}$(p-value). B) Shows the results with adjustment for p.C282Y genotype.

A)

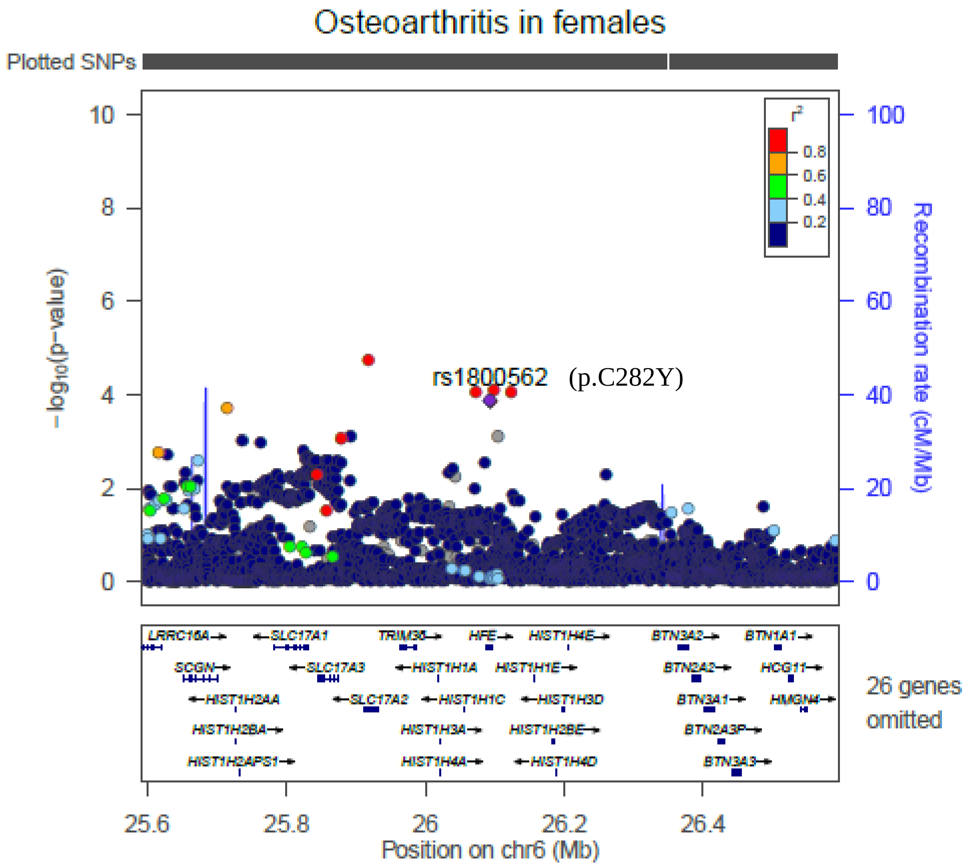

B)

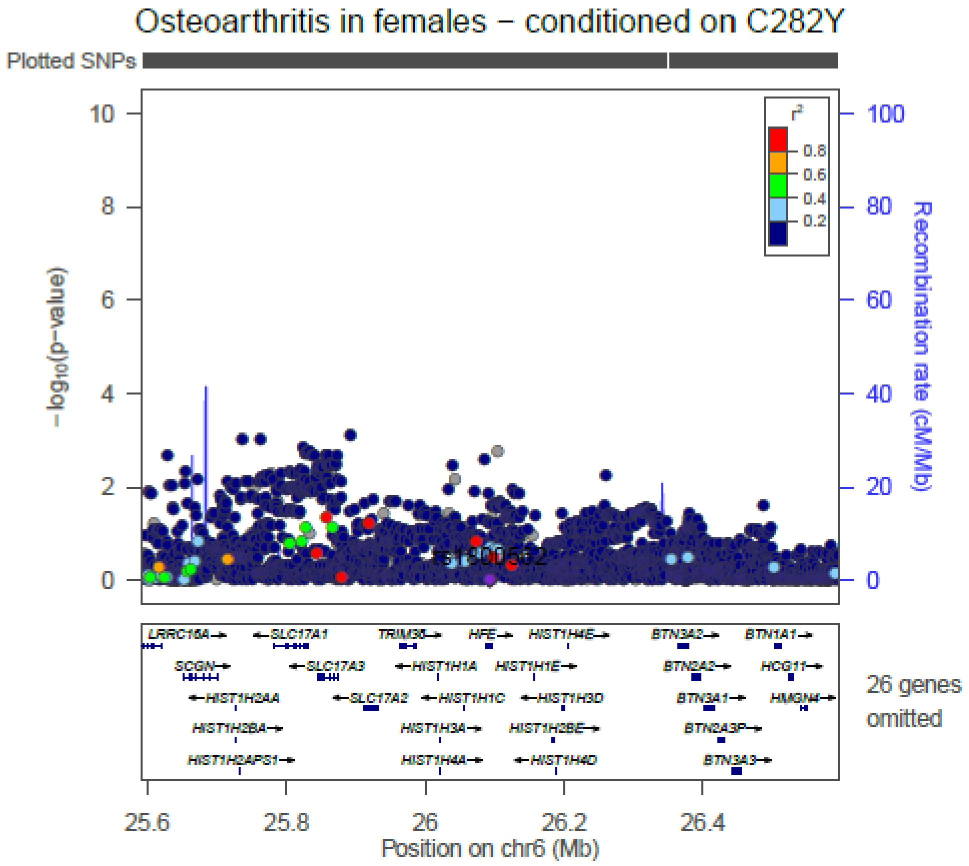

Supplementary Figure 26 – Colocalisation analysis of ≥1 prevalent diagnosis in females

A) LocusZoom plot of genotypes +/- 500kb of p.C282Y and their association with ≥1 diagnosis of haemochromatosis, CHD, diabetes, liver disease, osteoarthritis, osteoporosis, pneumonia, or Rheumatoid arthritis in females: y-axis = $-\log_{10}$(p-value). B) Shows the results with adjustment for p.C282Y genotype.

A)

B)

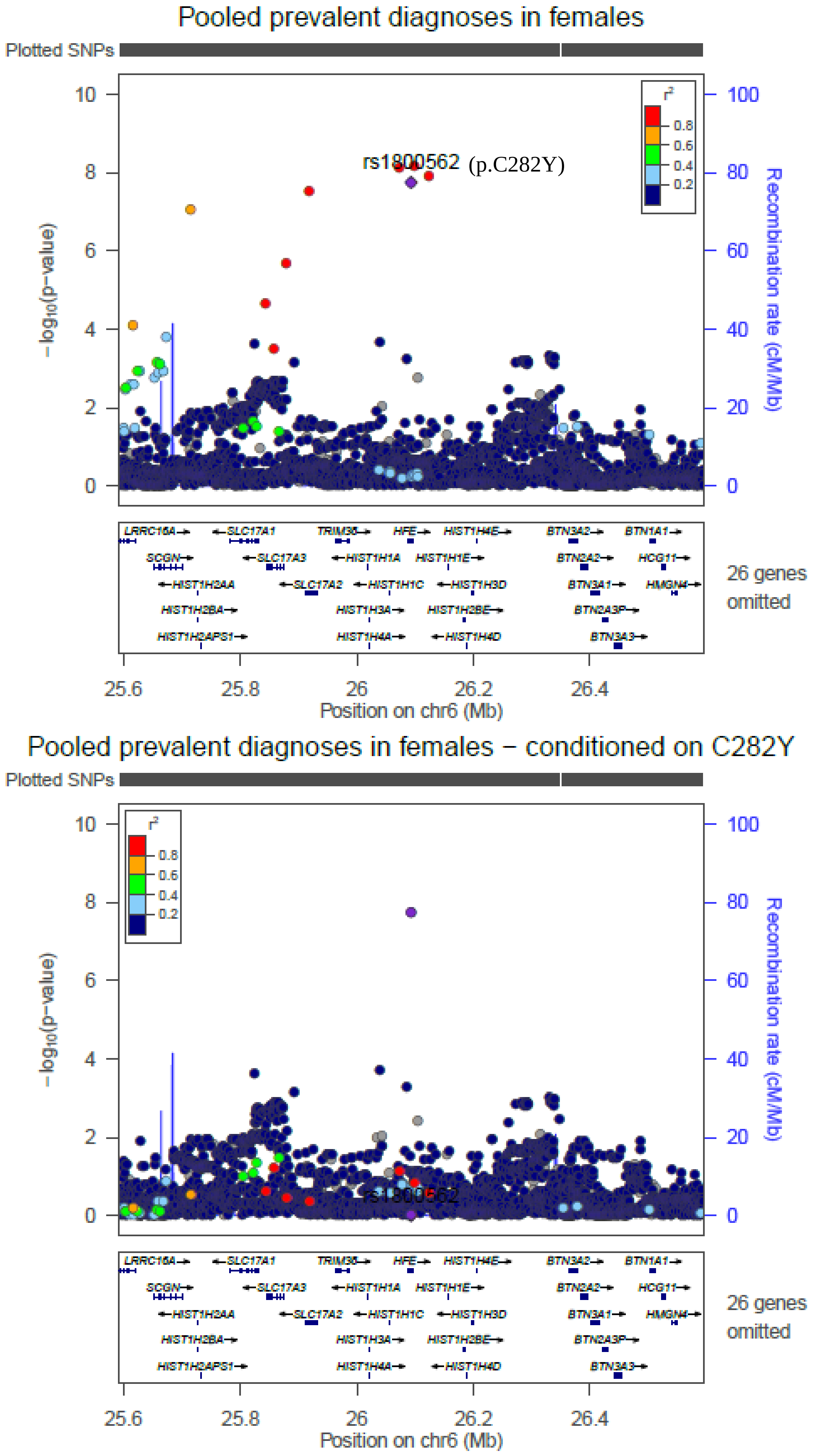

References

1. Bycroft C, Freeman C, Petkova D, et al. Genome-wide genetic data on ~500,000 UK Biobank participants. *bioRxiv*. July 2017.
2. Benyamin B, Esko T, Ried JS, et al. Novel loci affecting iron homeostasis and their effects in individuals at risk for hemochromatosis. *Nat Commun*. 2014;5(May):4926. doi:10.1038/ncomms5926.
3. Sudlow C, Gallacher J, Allen N, et al. UK biobank: an open access resource for identifying the causes of a wide range of complex diseases of middle and old age. *PLoS Med*. 2015;12(3):e1001779. doi:10.1371/journal.pmed.1001779.
4. Pruim RJ, Welch RP, Sanna S, et al. LocusZoom: regional visualization of genome-wide association scan results. *Bioinformatics*. 2010;26(18):2336-2337. doi:10.1093/bioinformatics/btq419.
5. Oexle K, Ried JS, Hicks AA, et al. Novel association to the proprotein convertase PCSK7 gene locus revealed by analysing soluble transferrin receptor (sTfR) levels. *Hum Mol Genet*. 2011;20(5):1042-1047. doi:10.1093/hmg/ddq538.
6. Raffield LM, Louie T, Sofer T, et al. Genome-wide association study of iron traits and relation to diabetes in the Hispanic Community Health Study/Study of Latinos (HCHS/SOL): potential genomic intersection of iron and glucose regulation? *Hum Mol Genet*. 2017;26(10):1966-1978. doi:10.1093/hmg/ddx082.
7. MacArthur J, Bowler E, Cerezo M, et al. The new NHGRI-EBI Catalog of published genome-wide association studies (GWAS Catalog). *Nucleic Acids Res*. 2017;45(D1):D896-D901. doi:10.1093/nar/gkw1133.
